# Cross-national harmonization of cognitive measures across HRS HCAP (USA) and LASI-DAD (India)

**DOI:** 10.1371/journal.pone.0264166

**Published:** 2022-02-25

**Authors:** Jet M. J. Vonk, Alden L. Gross, Andrea R. Zammit, Laiss Bertola, Justina F. Avila, Roos J. Jutten, Leslie S. Gaynor, Claudia K. Suemoto, Lindsay C. Kobayashi, Megan E. O’Connell, Olufisayo Elugbadebo, Priscilla A. Amofa, Adam M. Staffaroni, Miguel Arce Rentería, Indira C. Turney, Richard N. Jones, Jennifer J. Manly, Jinkook Lee, Laura B. Zahodne

**Affiliations:** 1 Department of Neurology, Taub Institute for Research on Alzheimer’s Disease and the Aging Brain, Vagelos College of Physicians and Surgeons, Columbia University, New York, New York, United States of America; 2 Julius Center for Health Sciences and Primary Care, University Medical Center Utrecht and Utrecht University, Utrecht, The Netherlands; 3 Department of Epidemiology, Center on Aging and Health, Johns Hopkins Bloomberg School of Public Health, Johns Hopkins University, Baltimore, Maryland, United States of America; 4 Rush Alzheimer’s Disease Center, Rush University Medical Center, Chicago, Illinois, United States of America; 5 Department of Psychiatry and Behavioral Sciences, Rush University Medical Center, Chicago, Illinois, United States of America; 6 Medical School, University of Sao Paulo, Sao Paulo, São Paulo, Brazil; 7 Alzheimer Center & Department of Neurology, Amsterdam UMC, Vrije Universiteit Amsterdam, Amsterdam Neuroscience, Amsterdam, the Netherlands; 8 Department of Clinical and Health Psychology, College of Public Health and Health Professions, University of Florida, Gainesville, Florida, United States of America; 9 Division of Geriatrics, University of Sao Paulo Medical School, Sao Paulo, São Paulo, Brazil; 10 Department of Epidemiology, Center for Social Epidemiology and Population Health, University of Michigan School of Public Health, Ann Arbor, Michigan, United States of America; 11 Department of Psychology, University of Saskatchewan, Saskatoon, Saskatchewan, Canada; 12 Department of Psychiatry, University of Ibadan, Ibadan, Nigeria; 13 Department of Clinical and Health Psychology, University of Florida, Gainesville, Florida, United States of America; 14 Department of Neurology, Memory and Aging Center, Weill Institute for Neurosciences, University of California at San Francisco (UCSF), San Francisco, California, United States of America; 15 Department of Psychiatry and Human Behavior, Warren Alpert Medical School, Brown University, Providence, Rhode Island, United States of America; 16 Center for Economic and Social Research & Department of Economics, Dornsife College of Letters, Arts, and Sciences, University of Southern California, Los Angeles, USA and RAND Corporation, Santa Monica, California, United States of America; 17 Department of Psychology, University of Michigan, Ann Arbor, Michigan, United States of America; University of Texas at Arlington, UNITED STATES

## Abstract

**Background:**

As global populations age, cross-national comparisons of cognitive health and dementia risk are increasingly valuable. It remains unclear, however, whether country-level differences in cognitive function are attributable to population differences or bias due to incommensurate measurement. To demonstrate an effective method for cross-national comparison studies, we aimed to statistically harmonize measures of episodic memory and language function across two population-based cohorts of older adults in the United States (HRS HCAP) and India (LASI-DAD).

**Methods:**

Data for 3,496 HRS HCAP (≥65 years) and 3,152 LASI-DAD (≥60 years) participants were statistically harmonized for episodic memory and language performance using confirmatory factor analysis (CFA) methods. Episodic memory and language factor variables were investigated for differential item functioning (DIF) and precision.

**Results:**

CFA models estimating episodic memory and language domains based on *a priori* adjudication of comparable items fit the data well. DIF analyses revealed that four out of ten episodic memory items and five out of twelve language items measured the underlying construct comparably across samples. DIF-modified episodic memory and language factor scores showed comparable patterns of precision across the range of the latent trait for each sample.

**Conclusions:**

Harmonization of cognitive measures will facilitate future investigation of cross-national differences in cognitive performance and differential effects of risk factors, policies, and treatments, reducing study-level measurement and administrative influences. As international aging studies become more widely available, advanced statistical methods such as those described in this study will become increasingly central to making universal generalizations and drawing valid conclusions about cognitive aging of the global population.

## Introduction

Several countries around the world conduct regular surveys to collect person-level microdata on health, socioeconomic status, retirement, and social networks in population-representative samples of their older populations [1]. With increasing burdens of cognitive impairment and dementia due to rapid global population aging, some of these large nation-wide studies have started to administer extensive cognitive assessments to a subset of their samples [2]. For example, the USA Health and Retirement Study (HRS) administered the Harmonized Cognitive Assessment Protocol (HCAP) to a random sample of their respondents aged 65+ in 2016 [3]. Mirroring the HCAP protocol, the Longitudinal Aging Study in India (LASI) administered the Diagnostic Assessment of Dementia (DAD) to a subset of their sample in 2017 [4]. Although the HRS-HCAP and LASI-DAD were intended to have comparable measures, these studies have methodological, administrative, and regional differences, which renders direct comparison challenging [5].

Harmonization of data entails efforts to combine data from multiple sources in a manner that they are suitable for comparison; statistical harmonization is a harmonization technique that uses a statistical process to convert scores on different variables across studies into common scales that can be used to directly compare across participants of the involved studies. Various methods for statistical harmonization exist, including standardization methods (e.g., T-scores and Z-transformations), multiple imputation models, and latent variable models [6]. Of these, latent variable models are among the preferred statistical harmonization methods, particularly because of the ability to incorporate heterogeneity due to sample characteristics and being the only statistical harmonization method in which measurement invariance can be examined [6].

Statistical harmonization is foundational work that can involve co-calibration of similar but not identical measurements across studies, allowing for direct and quantitative comparisons across datasets collected in different contexts, e.g., methodological variability or different languages of administration. Statistical harmonization of cognitive data from older adults across countries enables neuropsychological and epidemiological research that can address social, cultural, biological, medical, and demographic effects on cognitive aging and neurodegenerative diseases beyond the national scope. For example, statistical harmonization of the LASI-DAD sample with the HRS HCAP sample would make it possible to cross-nationally compare between the USA and India how life exposures, disparities, and risk factors contribute to cognitive aging and the risk of dementia. More specifically, statistical harmonization would allow to investigate questions about cross-national differences in the association of sex/gender and education (i.e., demographic factors) with episodic memory, or cross-national differences in the association of depressive symptoms of life course socio-economic status (i.e., risk factors) with cognition.

To successfully apply methods for statistical harmonization in cross-national research where there is no available sample in which all measures (or all versions of a particular measure) were given, it is imperative to establish that at least some tests measure the same underlying construct in the same way within each sample [7]. While tests may appear to be similar, cultural, social, linguistic, and racial/ethnic characteristics of participants may influence performance [8]. For example, a direct translation of a word-list learning test into a different language could tap different memory storage and retrieval processes as the selected words are highly prone to linguistic differences, such as word length and frequency [9]. More broadly defined, differential item functioning (DIF) is demonstrated when performance on a test item differs across groups of people with similar cognitive ability [10]. Evidence for DIF across groups is an important facet of measurement validity, but is under-examined in neuropsychology [11].

In addition to DIF, test information—directly related to the precision or marginal reliability of a factor—can vary over the range of performance. Test information may differ by study if studies have different numbers of test items and the items have varying levels of difficulty. Such a situation could interfere with cross-national comparisons by making it more likely to detect associations in the study with more precision. Moreover, if one study has more items, or systematically more, or less, difficult items than the other study, extreme scores cannot be reliably discriminated.

This study aimed to harmonize cross-national data of sister-studies on cognition in aging in the USA (HRS HCAP) and India (LASI-DAD). The specific objectives were to 1) describe the statistical harmonization process for cognitive domains in HRS HCAP and LASI-DAD with sufficient availability of comparable items (i.e., episodic memory and language), 2) identify items that measure the same underlying construct in the same way by testing and modifying for DIF across the two samples, 3) assess the precision of the scale in each study by investigating test information, and 4) present the resulting harmonized factor scores, their properties, and syntax for replication and application to other datasets.

## Methods

### Data sources

We harmonized data from two large cognitive aging studies: the HRS HCAP in the USA [3] and LASI-DAD in India [4]. The HRS is an ongoing nationally representative study on the health, economic, and social well-being of more than 43,000 adults aged 51 or older in the United States that began in 1992 [12, 13]. The HCAP is an HRS sub-study that aims to measure dementia risk using a parallel neuropsychological protocol administered in the HRS and several International Partner Studies [3]. A total of 3,496 individuals were randomly selected from HRS participants 65 and older who completed the 2016 core interview and venous blood collection [3, 12]. The one-hour respondent interview comprised cognitive measures (episodic memory, orientation, language, attention/executive functioning, working memory, processing speed, and fluid and crystallized intelligence), and the 20-minute informant interview comprised symptom perception and functional capacity measures [3]. Participants were evaluated in their preferred language, English or Spanish. Written consent was obtained from all HCAP participants and their informants, and the HRS and HCAP study protocols were approved by the University of Michigan Institutional Review Board.

LASI is an ongoing nationally representative survey on the health, economic, and social well-being of over 70,000 adults aged 45 years and over in 30 States and 6 Union Territories of India; the first wave of data collection was initiated in 2017 and completed in 2019 [4]. LASI is modeled after comparable studies in other countries, including the HRS [14, 15]. LASI-DAD builds on LASI’s initial cognitive assessment with a more detailed cognitive evaluation, including informant interviews. LASI participants across 14 States and Union Territories (*N* = 3,152) who were 60 years or older were selected for LASI-DAD [4]. LASI-DAD oversampled individuals at high risk of cognitive impairment [4]; sample weights were created that account for differential selection probabilities produced by the adopted sampling strategy and adjust for differential non-response. First, a design weight was computed to accounted for oversampling based on high risk of cognitive impairment. Using these design weights, a raking algorithm was applied to generate post-stratification weights. As such, the sample weights align the sample distributions of age and literacy, separately for men and women, and the distribution of rural versus urban residency to their population benchmarks as stated in the Indian Census 2011 for individuals aged 60 and above. The LASI-DAD cognitive assessment was based on the HRS HCAP protocol. Participants were evaluated in their local language, and the protocol was translated into 12 languages (Hindi, Kannada, Malayalam, Gujarati, Tamil, Punjabi, Urdu, Bengali, Assamese, Odiya, Marathi, and Telugu). Written consent was obtained from all participants and their informants, and the LASI and LASI-DAD protocols were approved by the Indian Council of Medical Research and all collaborating institutions.

### Cognitive measures

Both HCAP and LASI-DAD include a neuropsychological test battery measuring multiple cognitive domains. Instruments for episodic memory and language were taken from common examinations of global mental status, including the Consortium to Establish a Registry for Alzheimer’s Disease (CERAD) Word List and Praxis [16], Brave man story from the East Boston Memory Test [17], Logical Memory from the Wechsler Memory Scale Fourth Edition (WMS-IV) [18], Animal Fluency [16], and the Telephone Interview for Cognitive Status (TICS) [19]. The analyses were performed on raw test scores. LASI-DAD is representative of people age 60 and over in India and therefore many participants had low levels of literacy, requiring modification of several items [4]. Two modified items were included in the current study: *write a sentence* and *read and follow command*, which were administered to literate participants but replaced with *say a sentence* and *follow example (close your eyes)*, respectively, for illiterate participants. The majority of episodic memory items were continuous and the majority of language items categorical (Table 1).

**Table 1:** Overview of variables considered comparable in the *a priori* adjudication process and DIF-modified analyses summarized by cognitive domain, including their availability for each cohort.

			Variable	*A priori*	
Domain	Indicators	Source	type	adjudicated	Notes
*Memory*	Word list immediate recall	CERAD	Continuous	C	-
	Word list delayed recall	CERAD	Continuous	C	-
	Word list recognition	CERAD	Continuous	C	-
	Constructional praxis delayed recall	CERAD	Continuous	C	-
	Logical memory immediate recall	WMS	Continuous	C	-
	Logical memory delayed recall	WMS	Continuous	C	-
	Logical memory recognition	WMS	Continuous	C	-
	Brave man immediate recall	EBMT	Continuous	C	-
	Brave man delayed recall	EBMT	Continuous	C	-
	3-word delayed recall	MMSE	Categorical	C	-
*Language*	Animal fluency	WJIII	Continuous	C	-
	Name cactus	TICS	Categorical	-	HRS HCAP only
	Name coconut		Categorical	-	LASI-DAD only
	Name scissors	TICS	Categorical	C	-
	Name watch	MMSE	Categorical	C	-
	Name pencil	MMSE	Categorical	C	-
	Name elbow	CSI-D	Categorical	C	-
	Write a sentence	MMSE	Categorical	C	-
	Say a sentence		Categorical	-	LASI-DAD only
	Read and follow command	MMSE	Categorical	C	-
	Follow example		Categorical	-	LASI-DAD only
	Repetition of phrase	MMSE	Categorical	C	-
	What to do with a hammer	CSI-D	Categorical	C	-
	Where is the local market/store?	CSI-D	Categorical	C	-
	Following instructions 2 step	CSI-D	Categorical	C	-
	Following instructions 3 step	CSI-D	Categorical	C	-

*Note*. DIF = Differential Item Functioning; C = comparable item; Abbreviations: CERAD, Consortium to Establish a Registry for Alzheimer’s Disease; CSI-D, Community Screening Instrument for Dementia; EBMT, East Boston Memory Test; MMSE, Mini-mental state examination; TICS, Telephone Interview for Cognitive Status; WJIII, Woodcock-Johnson-III; WMS, Wechsler Memory Scale. ‘LASI-DAD only’ items are culturally- or illiteracy-adjusted items based on similar items from the provided test sources.

### Pre-statistical harmonization

Pre-statistical harmonization refers to the process of identifying relevant cognitive domains and instruments [6]. This process was done by reviewing study manuals and codebooks to determine whether test stimuli, administration procedures, scoring procedures, missing data handling, and response coding (e.g., possible minimum/maximum raw scores) are comparable across studies; selecting variables of interest for each cognitive instrument; and identifying candidate comparable items. Comparable items were identified as those that were judged to have been administered and scored similarly across studies. For the current study, an interdisciplinary team of neuropsychologists (LB, JF, RJ, LG, MO, AS, MAR, JM, LZ), psychometricians (AG, RJ), and a neurolinguist (JV) evaluated each available item. Cognitive items were categorized into cognitive domains, including episodic memory and language. Available data for each test item were reviewed for score ranges and distributions. Table 1 displays the variables identified to measure either episodic memory or language; of those, the items that were measured similarly in both studies were deemed comparable items. There were no items in these domains that were deemed not comparable in the pre-statistical harmonization process, which is likely attributable to the fact that HRS HCAP and LASI-DAD were designed as sister-studies on cognition in aging.

### Statistical approach

Participant characteristics across samples were analyzed using *t*-tests and chi-squared tests. The overall approach for statistical harmonization was to estimate a series of confirmatory factor analysis (CFA) models to develop co-calibrated factors for episodic memory and language based on all available items in these domains from HRS HCAP and LASI-DAD batteries. We defined HRS HCAP as the reference population and estimated a confirmatory factor analysis (CFA) for each cognitive domain. We saved the parameters from the HRS HCAP models and applied them to the comparable items in the LASI-DAD data (i.e., item-banking approach) and estimated parameters for unique LASI-DAD items. A final score-generating model per domain pooled all HRS HCAP and LASI-DAD participants using all previously estimated parameters. These steps are described in more detail in the paragraphs below.

We first estimated a confirmatory factor analysis (CFA) for each domain in HRS HCAP using all available items in HRS HCAP for the domain (S1 File, non-DIF modified models, Step 1 for episodic memory and language). Mean and variance of the factor (episodic memory or language) were set to 0 and 1, respectively, for model identification. Model fit was ascertained using standard absolute fit statistics, including the Root Mean Square Error of Approximation (RMSEA, good fit < .06), Comparative Fit Index (CFI, good fit >.95), and Standardized Root Mean Residual (SRMR, good fit < .08) [20]. For the language domain, the CFA model was best fitted with a unidimensional structure. For the episodic memory domain, a bifactor CFA model provided best fit, which accounted for additional covariance among scores from different trials of the same test: Logical Memory Test, Brave Man test, and Word List.

For each cognitive test item, the CFA model estimated two sets of parameters. First, factor loadings described how well an item separated people of low and high ability on the latent trait (episodic memory or language), or equivalently, how strongly the item was correlated with other tests measuring the trait. In general, factor loadings larger than .30 indicate an item is meaningfully related to the underlying latent trait, but criteria for loadings must also depend on theoretical considerations [21]. Second, thresholds, or boundaries, for categorical items, or intercepts, or levels, for continuous items described the location along the range of the latent trait where the probability of responding with a given performance level or better is 50%. For example, easier test items are those on which a higher proportion of the sample performed well, and more difficult test items are those on which a lower proportion of the sample performed well. These parameters from the first CFA models (i.e., item factor loadings and threshold or intercept parameters) were saved for use in the subsequent steps.

After estimating CFAs for each domain in HRS HCAP, we estimated a second round of CFAs for each domain among participants in LASI-DAD, in which parameters (loadings and thresholds/intercepts) of comparable items were constrained to what they were in the HRS HCAP models. Of particular concern were the LASI-DAD language items *write a sentence* and *read and follow command;* these items were modified for administration to illiterate individuals. Therefore, we decided *a priori* to consider the LASI-DAD sample as two samples (literate vs. illiterate) for co-calibrating the language factor. Parameters for unique items in LASI-DAD that were not in HRS HCAP were freely estimated, as were means and variances of the episodic memory and language factors (S1 File, non-DIF modified models, Step 2 for episodic memory and Steps 2/3 for language).

In the final score-generating model for each domain, we pooled all participants per domain (for episodic memory from the HRS HCAP and LASI-DAD models, and for language from the HRS HCAP model and LASI-DAD models for literate and illiterate participants) to estimate one CFA for each domain in which all item parameters were fixed to their previously estimated values and no parameters were freely estimated (S1 File, non-DIF modified models, Step 3 for episodic memory and Step 4 for language). These models produced the non-DIF modified episodic memory and language factor score estimates.

**Fig 1:**
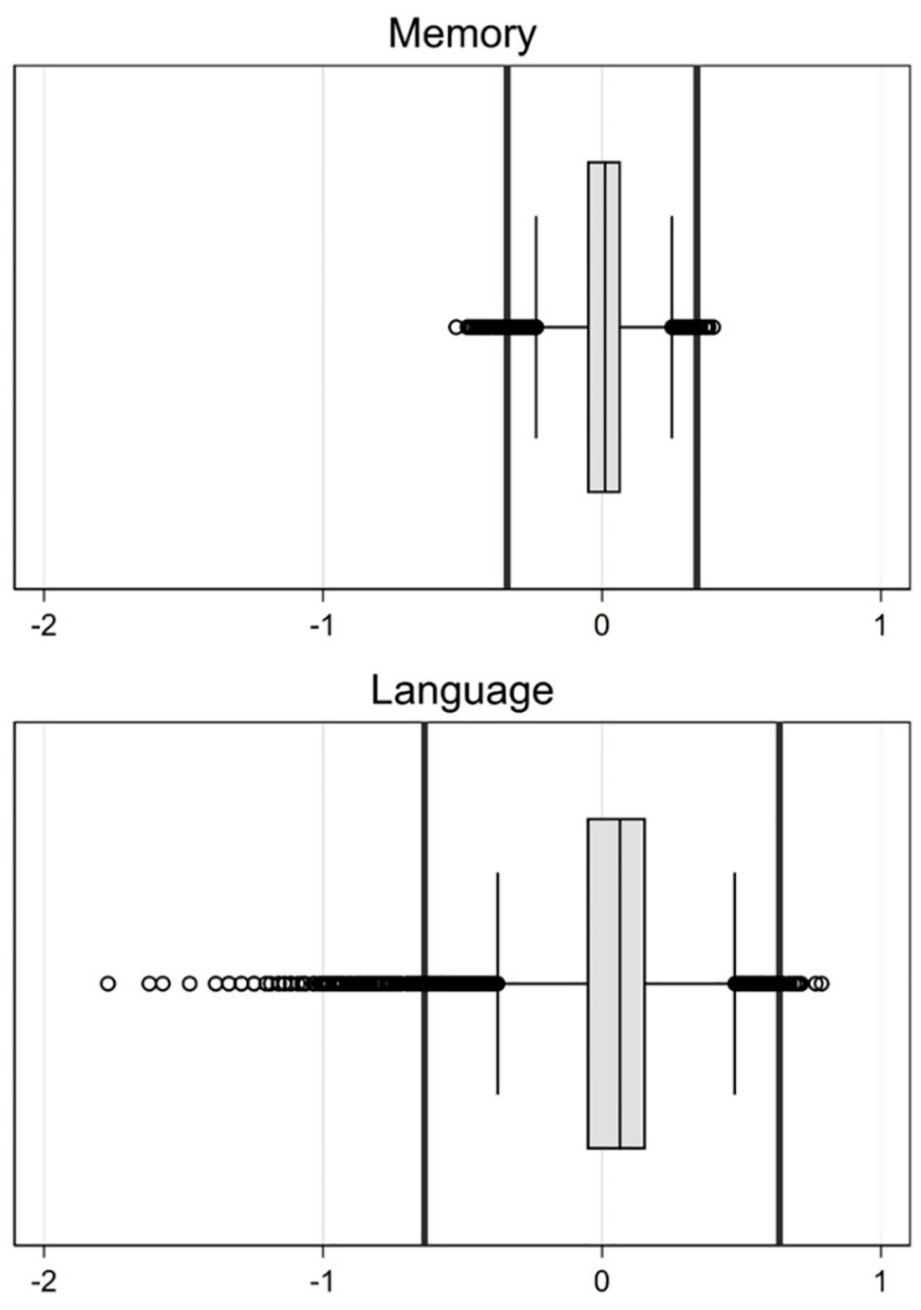
Differential Item Functioning (DIF) impact; the boxplots represent the difference scores of initial and DIF-modified scores per domain and the vertical lines represent 1x the standard error of measurement of the sample, i.e., the threshold for salient DIF.

**Fig 2:**
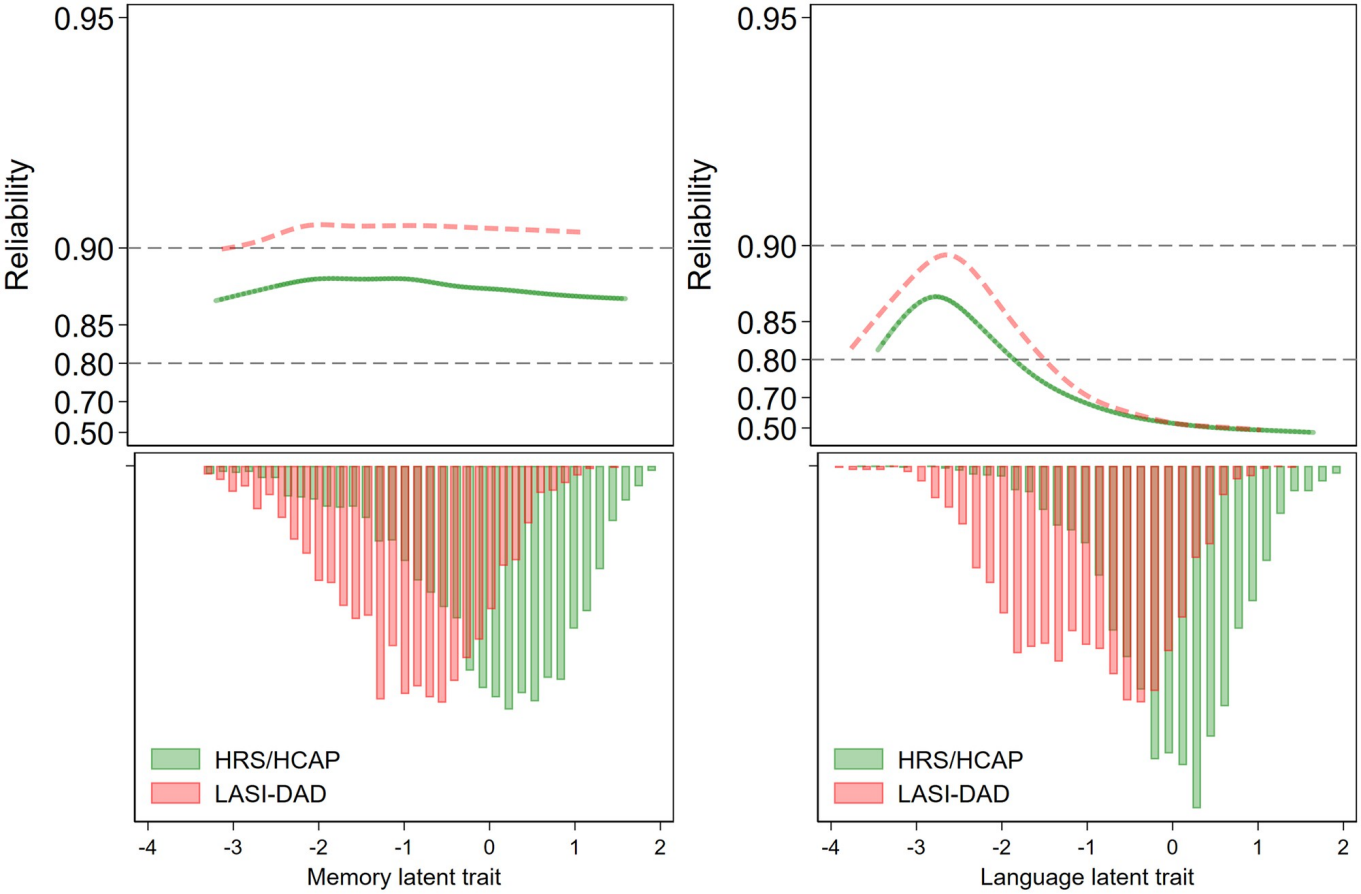
Information curves for the Differential Item Functioning (DIF)-modified episodic memory and language factors (reliability = 1–1/information) (upper panel). The histograms are the population distribution on the latent trait (lower panel). With mostly continuous factor indicators for the episodic memory latent trait, reliability is constant over the range of theta (ability). With mostly categorical indicators for the language latent trait, reliability varies over the range of theta, as shown by a peak where most of the item difficulty parameters are.

We then evaluated and modified the scores for DIF attributable to study, applying ordinal logistic regression for categorical variables and linear regression for continuous variables (Fig 1; Table 4; S2 File) [22]. This regression approach allowed for adjustment by age, sex, and years of education. DIF detection using regression entails estimating a series of regression models of each item on the factor score for the cognitive domain (model 1), and on the factor score and an indicator for study (model 2). Likelihood ratio tests of models 1 and 2 test for DIF in thresholds or intercepts for a given item at a threshold of p < .05 for significant difference [22]. The regression approach requires a fixed and presumed error-free estimate of a factor score from a model assuming no DIF; the CFA model was re-estimated after each iteration for DIF detection allowing the parameter to vary first for the item with the highest likelihood ratio test value (S1 File, DIF modified models for episodic memory and language). We tested for uniform DIF, which assumes that, on average, performance on an item is consistently more difficult for one group than another at similar levels of ability. We did not investigate non-uniform DIF, in which differential performance interacts with level of abilities and group membership, because it is challenging to distinguish uniform from non-uniform DIF in this particular context; uniform DIF should be expected when there is non-uniform DIF, if item location parameters are far away from the mean level of the underlying latent trait in at least one sample, which is the case for HRS HCAP vs. LAS-DAD. After evaluating evidence of uniform DIF and arriving at final models, we compared test information curves derived from these final DIF-modified models between HRS HCAP and LASI-DAD (Fig 2). We also determined whether observed DIF was “salient”, i.e., whether an individual’s DIF-modified score was considerably different—as measured by ≥1 standard error of measurement—from their initial score [22].

CFA models were estimated with Mplus software (Version 8.2, Muthén & Muthén, Los Angeles CA). Stata software (Version 16.1, Stata Corp, College Station, TX) was used for data management, DIF detection using regression, and generation of item information curves. Syntax is provided in S1 and S2 Files.

## Results

### Sample characteristics

Demographic descriptive statistics for each study and mean performance on cognitive test items are in Table 2. Compared to the HRS HCAP sample, the LASI-DAD sample was younger, had a higher percentage of men, had fewer years of education, and performed worse on all cognitive tests except on the *write a sentence* item. For example, the HRS HCAP sample recognized on average 18.5 out of 20 words on word list recognition and the LASI-DAD sample 16 words as part of the episodic memory tasks; as an example of language tasks, the HRS HCAP sample generated on average 16 words on animal fluency (i.e., naming as many animals during one minute), while the LASI-DAD sample generated on average 12 words. These differences persisted when stratifying the LASI-DAD participants by literacy: comparing the HRS HCAP participants with the literate LASI-DAD participants only, the latter were still younger, had a higher percentage of men, had fewer years of education, and performed worse on all cognitive tests except on the items *name a watch* and *write a sentence*. Matrices of correlations between items for each cohort are presented in S3 File.

**Table 2:** Sample characteristics for HRS HCAP and LASI-DAD (overall and stratified by literacy).

		HRS HCAP (*n* = 3496)	LASI-DAD (*n* = 3152)	LASI-DAD literate (*n* = 1403)	LASI-DAD illiterate (*n* = 1749)
		m (SD; range)/n (%)	m (SD; range)/n (%)	m (SD; range)/n (%)	m (SD; range)/n (%)
Age		76.6 (7.5; 65–102)	69.3 (7.7; 60–104)	68.8 (7.2; 60–100)	69.8 (8.1; 60–104)
Women		2095 (60%)	1693 (53.7)	522 (37%)	1171 (67%)
Education (years)		12.7 (3.2; 0–17)	4.0 (4.7; 0–21)	7.9 (4.2; 0–21)	.9 (2.2; 0–20)
Literate		-	1749 (55%)	1403 (100%)	1749 (0%)
Lives in rural area		64 (2%)	1913 (60.7)	682 (49%)	1231 (70%)
Language of administration	English	3312 (95%)	10 (.3%)	9 (.6%)	1 (.1%)
	Spanish	178 (5%)	-	-	-
	Hindi	-	983 (31%)	328 (23%)	655 (37%)
	Kannada	-	244 (8%)	100 (7%)	144 (8%)
	Malayalam	-	349 (11%)	269 (19%)	80 (5%)
	Tamil	-	299 (10%)	148 (11%)	151 (9%)
	Urdu	-	152 (5%)	26 (2%)	126 (7%)
	Bengali	-	294 (9%)	142 (10%)	152 (9%)
	Assamese	-	199 (6%)	93 (7%)	106 (6%)
	Odiya	-	252 (8%)	124 (9%)	128 (7%)
	Marathi	-	181 (6%)	102 (7%)	79 (5%)
	Telugu	-	189 (6%)	62 (4%)	127 (7%)
*Episodic memory* (mean or proportion correct)	Word list immediate recall	17.45 (5.20; 0–30)	11.43 (5.19; 0–28)	13.66 (4.75; 0–28)	9.63 (4.83; 0–24)
	Word list delayed recall	5.12 (2.65; 0–10)	3.13 (2.36; 0–10)	3.99 (2.31; 0–10)	2.44 (2.16; 0–9)
	Word list recognition	18.49 (2.48; 0–20)	15.98 (3.58; 0–20)	17.43 (2.76; 3–20)	14.81 (3.74; 0–20)
	Constructional praxis delayed recall	5.81 (3.24; 0–11)	2.72 (2.66; 0–11)	3.98 (2.90; 0–11)	1.71 (1.92; 0–11)
	Logical memory immediate recall	9.83 (5.10; 0–23)	4.05 (4.14; 0–24)	5.47 (4.58; 0–24)	2.82 (3.26; 0–18)
	Logical memory delayed recall	7.39 (5.39; 0–25)	3.14 (4.26; 0–25)	4.78 (4.78; 0–25)	1.66 (3.05; 0–21)
	Logical memory recognition	10.28 (2.74; 0–15)	7.72 (3.11; 0–15)	8.79 (2.74; 0–15)	6.80 (3.12; 0–14)
	Brave man immediate recall	7.11 (2.44; 0–12)	5.28 (3.13; 0–12)	6.38 (2.94; 0–12)	4.35 (2.98; 0–12)
	Brave man delayed recall	5.09 (3.30; 0–12)	2.92 (3.51; 0–12)	4.29 (3.76; 0–12)	1.77 (2.80; 0–12)
	3-word delayed recall	2.54 (0.76; 0–3)	1.94 (1.07; 0–3)	2.15 (0.97; 0–3)	1.77 (1.12; 0–3)
*Language* (mean or proportion correct)	Animal fluency	15.97 (6.57; 0–43)	11.78 (4.91; 0–70)	13.42 (4.92; 0–60)	10.46 (4.48; 0–70)
	Name cactus	0.97 (0.17; 0–1)	-	-	-
	Name coconut	-	0.58 (0.49; 0–1)	0.71 (0.45; 0–1)	0.48 (0.50; 0–1)
	Name scissors	0.99 (0.12; 0–1)	0.82 (0.38; 0–1)	0.88 (0.33; 0–1)	0.78 (0.41; 0–1)
	Name watch	1.00 (0.07; 0–1)	0.98 (0.15; 0–1)	0.99 (0.09; 0–1)	0.96 (0.19; 0–1)
	Name pencil	0.99 (0.08; 0–1)	0.86 (0.35; 0–1)	0.93 (0.25; 0–1)	0.80 (0.40; 0–1)
	Name elbow	0.99 (0.09; 0–1)	0.94 (0.23; 0–1)	0.97 (0.18; 0–1)	0.92 (0.27; 0–1)
	Write a sentence	0.94 (0.24; 0–1)	0.93 (0.25; 0–1)	0.93 (0.25; 0–1)	-
	Say a sentence	-	0.83 (0.38; 0–1)	-	0.83 (0.38; 0–1)
	Read and follow command	0.97 (0.16; 0–1)	0.43 (0.49; 0–1)	0.43 (0.49; 0–1)	-
	Follow example	-	0.83 (0.38; 0–1)	-	0.83 (0.38; 0–1)
	Repetition of phrase	0.70 (0.46; 0–1)	0.89 (0.31; 0–1)	0.95 (0.21; 0–1)	0.84 (0.37; 0–1)
	What to do with a hammer	0.93 (0.26; 0–1)	0.73 (0.44; 0–1)	0.82 (0.38; 0–1)	0.66 (0.47; 0–1)
	Where is the local market/store?	0.84 (0.37; 0–1)	0.90 (0.30; 0–1)	0.95 (0.21; 0–1)	0.86 (0.35; 0–1)
	Following instructions 2 step	0.99 (0.10; 0–1)	0.89 (0.32; 0–1)	0.93 (0.25; 0–1)	0.85 (0.36; 0–1)
	Following instructions 3 step	2.76 (0.47; 0–3)	2.62 (0.69; 0–3)	2.79 (0.51; 0–3)	2.49 (0.78; 0–3)

*Note*. m = mean, SD = standard deviation;— = not administered.

### Episodic memory

The episodic memory CFA fit well in the first step using only HRS HCAP data and freely estimating all item parameters (RMSEA = .059; CFI = .962; SRMR = .023). Standardized factor loadings of the final model, based on the step-wise estimation from the CFA for the HRS HCAP sample and the CFA for the LASI-DAD sample, ranged between .59 and .82 (Table 3).

**Table 3:** Standardized factor loadings and thresholds or intercepts for episodic memory and language from the non-modified and DIF-modified CFA models.

		Not modified for DIF			DIF-modified			
		Factor loading		Threshold or intercept		Factor loading		Threshold or intercept
*Episodic memory*	Word list immediate recall	0.80		3.36	*HRS HCAP*	0.80		3.36
					*LASI-DAD*	0.84		2.85
	Word list delayed recall	0.82		1.94		0.82		1.94
	Constructional praxis delayed recall	0.65		1.79	*HRS HCAP*	0.65		1.79
					*LASI-DAD*	0.57		1.49
	Word list recognition	0.71		7.82	*HRS HCAP*	0.71		7.82
					*LASI-DAD*	0.88		5.48
	Logical memory immediate recall	0.69		1.93	*HRS HCAP*	0.69		1.93
					*LASI-DAD*	0.61		1.49
	Logical memory delayed recall	0.73		1.38		0.73		1.38
	Logical memory recognition	0.60		3.75	*HRS HCAP*	0.60		3.75
					*LASI-DAD*	0.70		3.23
	Brave man immediate recall	0.59		2.92		0.59		2.92
	Brave man delayed recall	0.63		1.54		0.63		1.54
	3-word delayed recall	0.72	1	-1.86	*HRS HCAP*	0.72	1	-1.86
			2	-1.30			2	-1.30
			3	-0.44			3	-0.44
					*LASI-DAD*	0.59	1	-1.59
							2	-1.06
							3	-0.35
*Language*	Animal fluency	0.57		2.43		0.62		2.43
	Name cactus	0.64		-1.88		0.67		-1.88
	Name coconut	0.48		-1.14		0.60		-1.09
	Name scissors	0.56		-2.21	*HRS HCAP*	0.58		-2.21
					*LASI-DAD*	0.43		-1.53
	Name watch	0.79		-2.59		0.82		-2.59
	Name pencil	0.67		-2.46		0.69		-2.46
	Name elbow	0.83		-2.43		0.86		-2.43
	Write a sentence	0.58		-1.56	*HRS HCAP*	0.60		-1.56
					*LASI-DAD*	0.73		-1.94
	Say a sentence	0.77		-2.08		0.81		-2.16
	Read and follow command	0.84		-1.93	*HRS HCAP*	0.52		-1.93
					*LASI-DAD*	0.52		-0.35
	Follow example	0.79		-2.10		0.84		-2.19
	Repetition of phrase	0.46		-0.54	*HRS HCAP*	0.48		-0.54
					*LASI-DAD*	0.58		-2.07
	What to do with a hammer	0.34		-1.46		0.36		-1.46
	Where is the local market/store?	0.43		-0.98	*HRS HCAP*	0.45		-0.98
					*LASI-DAD*	0.62		-2.09
	Following instructions 2 step	0.81		-2.35	*HRS HCAP*	0.82		-2.35
					*LASI-DAD*	0.79		-1.95
	Following instructions 3 step	0.57	1	-3.12	*HRS HCAP*	0.37	1	-3.12
			2	-2.15			2	-2.15
			3	-0.76			3	-0.76
					*LASI-DAD*	0.53	1	-2.82
							2	-2.18
							3	-1.41

*Note*. For the language domain, two items were only administered among literate participants (Write a sentence, Read and follow command) and two were substituted for illiterate participants (Say a sentence, Follow example). As described in the Methods, this was handled by first estimating model parameters among literate participants, then estimating another model among illiterate participants with item parameters fixed to the model using literate participants. DIF = Differential Item Functioning; CFA = confirmatory factor analysis.

The DIF analysis showed that four candidate items could be considered comparable items for episodic memory—Logical memory delayed recall, Brave man immediate recall, Brave man delayed recall, and Word list delayed recall—while it detected the presence of DIF in six items (Table 4). For example, a regression model to detect DIF in which the relationship between performance on immediate recall of a word list and the episodic memory factor score was not adjusted for study differed from a regression model in which this relationship was adjusted for study, an indication of DIF. In contrast, similar regression models for the relationship between performance on immediate recall of the Brave man story did not differ with or without an indicator for study in the model, indicating no DIF for this item. We re-estimated the CFA model to obtain DIF-modified episodic memory factor scores (Table 3). The salient DIF results suggested that only .8% of episodic memory scores (n = 51) were considerably different—by at least 1 standard error of measurement—once we modified for observed DIF, indicating negligible DIF impact (Fig 1).

**Table 4:** DIF detection using logistic and linear regression.

Cognitive test and domain	Logistic or linear regression		DIF
	Estimate^1^	Chi-square	
Episodic memory			
Word list immediate recall, b (SE)	-1.14 (0.10)	118.95	Yes
Word list delayed recall, b (SE)	-0.29 (0.05)	35.13	No
Word list recognition, b (SE)	-0.53 (0.08)	49.41	Yes
Constructional praxis delayed recall, b (SE)	-1.02 (0.08)	157.88	Yes
Logical memory immediate recall, b (SE)	-1.04 (0.09)	140.88	Yes
Logical memory delayed recall, b (SE)	-0.50 (0.09)	32.67	No
Logical memory recognition, b (SE)	-0.55 (0.07)	55.72	Yes
Brave man immediate recall, b (SE)	0.03 (0.07)	0.25	No
Brave man delay, b (SE)	0.14 (0.08)	2.99	No
3-word delayed recall, OR (SE)	-0.57 (0.07)	70.03	Yes
Language			
Animal fluency, b (SE)	-0.96 (0.18)	27.45	No
Name scissors, OR (SE)	-2.06 (0.22)	104.96	Yes
Name watch, OR (SE)	-0.40 (0.49)	0.68	No
Name pencil, OR (SE)	-1.67 (0.29)	33.79	No
Name elbow, OR (SE)	-1.22 (0.35)	12.55	No
Write a sentence, OR (SE)	1.34 (0.21)	46.57	Yes
Read and follow command, OR (SE)	-3.70 (0.17)	702.39	Yes
Repetition of phrase, OR (SE)	2.59 (0.14)	437.41	Yes
Where is the local market/store, OR (SE)	1.91 (0.16)	170.16	Yes
What to do with a hammer, OR (SE)	-0.81 (0.14)	33.31	No
Following instructions 2 step, OR (SE)	-1.67 (0.27)	41.46	Yes
Following instructions 3 step, OR (SE)	0.84 (0.11)	57.634	Yes

*Note*. Reference group is HRS HCAP; B = regression parameter estimate (unstandardized), OR = odds ratio; DIF = Differential Item Functioning;

^1^The beta coefficient is the difference in an item mean or threshold between LASI-DAD and HRS/HCAP.

Plotting measurement precision across HRS HCAP and LASI-DAD showed that the episodic memory factor maintained high precision throughout the range of the latent trait in both samples, yet slightly higher in LASI-DAD than HRS HCAP (Fig 2). This pattern is consistent with marginally higher factor loadings for many episodic memory items in LASI-DAD compared to HRS HCAP (Table 3). Moreover, the episodic memory factor showed a comparable pattern of precision along the latent trait range for each study.

### Language

The language factor fit moderately in the first step using only HRS HCAP data and freely estimating all item parameters (RMSEA = .014; CFI = .980; SRMR = .088). Standardized factor loadings of the final model, based on the step-wise estimation from the CFA for the HRS HCAP sample, the CFA for the LASI-DAD literate sample, and the CFA for the LASI-DAD illiterate sample, ranged between .34 and .84.

The DIF analysis showed that only five items could be considered comparable items—animal fluency, *name a watch*, *name a pencil*, *name an elbow*, and *what to do with a hammer*—while evidence for DIF was found for seven items (Table 4). The CFA model to obtain the language factor score was re-estimated with DIF modification (Table 3). The salient DIF results suggested that 6.7% of the DIF-modified language scores (n = 445, of whom n = 423 were from the LASI-DAD sample) differed from the initial scores by at least 1 standard error of measurement. This result indicates considerable DIF impact on the language scores, particularly among LASI-DAD participants (Fig 1).

Plotting measurement precision of the language factor across HRS HCAP and LASI-DAD showed that the factor has higher precision at lower levels of underlying language ability compared to higher levels in each study (Fig 2). It is notable that this higher precision occurs at a location on the latent trait that represents a relatively low number of participants that have this lower level of underlying language ability on the latent trait.

## Discussion

The ability of neurocognitive assessments to evaluate cognitive domains equivalently across demographically different cohorts is essential; it allows for parallel analysis while identifying individual factors responsible for observed differences. This study harmonized episodic memory and language ability estimates across two large national cognitive aging studies in the USA (HRS HCAP) and India (LASI-DAD). Because DIF analyses revealed that the majority of *a priori-*deemed comparable episodic memory and language items were statistically different, DIF-modified factor scores are critical for future studies seeking to combine or compare data from HRS HCAP and LASI-DAD. Both DIF-modified factors showed a comparable pattern of measurement precision along the latent trait range for each study.

Our interdisciplinary author team thought that certain items would be statistically comparable across studies, controlling for underlying episodic memory or language ability, but we also empirically tested whether this assumption was the case. Although 22 possible comparable items were identified from the pre-statistical harmonization, our analyses showed that only four out of ten episodic memory items and five out of twelve language items measured the underlying construct the same way across cohorts. LASI-DAD measures were translated and adapted from the English-language HCAP measures into 12 languages, with culturally appropriate modifications [4]. While the translation of English-language tests provides rich data for cross-national comparisons, the direct translation of measures does not ensure the equivalence of different language versions across and within cultures and countries [23]. While recent work suggested minimal differences overall by language of administration within LASI-DAD [24], future research should investigate DIF by language of administration within the language domain separately: translation artifacts, including cross-language differences in idiomatic expressions, terminology, and nomenclature may alter the difficulty level of language items in particular [25]. Evidence for DIF in multiple episodic memory and language tests underscores the importance of evaluating the extent to which items may be measuring different abilities across groups of participants, a currently under-examined practice in neuropsychology [11]. A strength of this study includes using a regression approach for DIF analyses, which allows adjusting for individual differences in age, sex, and years of education. As such, the detected DIF is likely due to study-specific differences after adjusting for these individual differences. Moreover, we also determined whether the individual-level DIF impact was salient: we showed that once we modified for observed DIF, the DIF impact on episodic memory scores was negligible while the DIF impact on language scores was considerable, particularly among LASI-DAD participants. We recommend that other cross-national studies also undertake these steps and make DIF-modified harmonized scores available to minimize bias in cross-national comparisons, to ensure that we truly are measuring the same construct in the same way across groups.

While the test information curve for episodic memory showed relatively equal precision across the latent trait range for both samples, which is desirable, precision for episodic memory was slightly higher in the LASI-DAD than HRS HCAP sample. Comparison of loadings for HRS HCAP episodic memory items to those for LASI-DAD episodic memory items revealed that the items have less variability due to the apparent ceiling effect in HRS HCAP, and thus less variance to share with other items. This effect leads to systematically lower episodic memory factor loadings in HRS HCAP than in LASI-DAD. Thus, the systematically higher mean performance of HRS HCAP participants than LASI-DAD participants on episodic memory items likely resulted in these items providing less information about the episodic memory ability of HRS HCAP participants compared to those in the LASI-DAD sample. However, this difference in precision was relatively small and the factor maintained high precision in both samples.

For language, the test information curves were more similar across countries, but the precision of the language factor was increased at lower levels of language ability in both samples. This pattern may reflect that many of the language items were taken from aphasia batteries that were designed to measure linguistic skills among people with moderate to severe language impairment. Moreover, this pattern may be influenced by the relatively low number of participants that have this lower level of underlying language ability on the latent trait. The implication of the low reliability for the language domain suggests that these items are not optimal for research in community settings. For example, one of the language items had a factor loading of .34, meaning that only 12% of the variance on the item reflected underlying language ability. A future challenge for our field will be to implement language measures that can assess different linguistic skills across diverse settings around the world. The analyses of test information facilitate assessment of the precision or marginal reliability with which latent traits were measured over the range of performance. However, this analysis does not allow for inferences about the type of respondents in each population that the scales can reliably distinguish. It is conceivable that certain participant characteristics might drive test performance, and this is an important course for future research.

Harmonization is a critical first step in understanding factors driving cross-national differences in cognitive impairment. Within-country differences in cognitive function, decline, and dementia risk at older age have previously been observed across sex/gender, race/ethnicity, urban-rural residence, and life-course socioeconomic status indicators, including education, income, and employment [26–28]. In addition to the harmonization of measures, differences in sampling strategies and sample composition need to be carefully taken into account when interpreting between-country differences in cognitive ability and effects of predictors.

Harmonization is also required to understand cross-national differences in disparities. Differences in socioeconomic status within the US are on a different scale from comparisons of the US with India and other low and middle income countries. Because HRS HCAP and LASI-DAD cognitive batteries were successfully harmonized, cross-national differences in the magnitude of inequalities in cognitive function across SES may provide new opportunities to investigate life-course risk and resilience factors for cognitive aging and the risk of dementia. Our harmonized factor scores can be used by other researchers to explore differences in memory and language performance across the US and India. Harmonization of cognitive measures will facilitate future investigation of cross-national differences in cognitive performance and differential effects of risk factors, policies, and treatments, reducing study-level measurement and administrative influences. We have provided syntax for replication and application to other datasets in the S1 and S2 Files.

Our harmonization effort was limited by the cognitive items that were included in each battery; the inclusion of more sensitive tests from the same domains or tests from other cognitive domains would have presented different challenges [8]. Advanced harmonization techniques may be needed to include executive functioning and processing speed tests, which did not have sufficient comparable items for the methods that we used in this analysis. Undocumented variations in the administration and scoring of tests are possible, but were beyond our control and could not be accounted for during the pre-statistical harmonization process. We were unable to pinpoint whether the detected DIF was due to cultural/geographical differences, language differences, administrative differences, recruitment differences, or methodological differences across HRS HCAP and LASI-DAD. While the analyses *harmonized* the episodic memory and language domains, they have not been *equated*, as shown by the unequal precision of the episodic memory factor across the HRS HCAP and LASI-DAD samples. This difference in precision may introduce bias in country-level comparisons of episodic memory ability; a simulation study would be required to investigate the presence and magnitude of such bias.

The importance of harmonizing cognitive measures and testing for measurement equivalence is an essential part of cross-national comparisons [29]. Statistical harmonization techniques can improve the comparability of cross-national datasets to address the social, cultural, biological, and environmental factors that affect normal and abnormal cognitive aging, including the risk of Alzheimer’s disease and other dementias. As data from international aging studies become more widely available, harmonization of cognitive measures supports cross-national collaborations that will enhance the generalizability, applicability, and validity of cognitive aging research.

## Supporting information

S1 File Mplus syntax for statistical harmonization. (DOCX)

S2 File Stata syntax for DIF analyses with regression approach. (DOCX)

S3 File Correlation matrices of items for HRS HCAP and LASI-DAD. (DOCX)
