# Supplementary material for: Cross-national harmonization of cognitive measures across HRS HCAP (USA) and LASI-DAD (India)
Source: PLoS One. 2022 Feb 25;17(2):e0264166. doi: 10.1371/journal.pone.0264166 (PMC8880818; doi:10.1371/journal.pone.0264166)
Supplement: S1 File — (DOCX) [file pone.0264166.s001.docx]

# Memory domain

## Memory factor: Non-DIF adjusted

Mplus input files for each of the following models (item banking approach):

mem_bnk1 - Memory CFA in HRS/HCAP (step 1)

mem_bnk3 - Memory CFA in LASI-DAD (step 2)

mem_bnk4 - Memory CFA in full sample (step 4) - 0 parameters are estimated, factor scores are estimated from this model

### Step 1

TITLE:

Variable List -

u3 : CERAD immediate sum of 3 trials

u5 : LM immediate

u7 : Brave man immediate

u8 : Brave man delay

u9 : LM delay

u11 : MMSE 3-word delay

u12 : CERAD word list delay

u14 : CERAD constructional praxis delay (MexCog = 4 figures)

u15 : CERAD recognition

u17 : LM recognition

idunique :

dat :

0: 0 HCAP

DATA:

FILE = mem_bnk1.dat ;

VARIABLE:

NAMES =

u3 u5 u7 u8 u9 u11 u12 u14 u15 u17 idunique dat ;

MISSING ARE ALL (-9999) ;

idvariable=idunique ;

auxiliary=dat ;

categorical= u11 ;

ANALYSIS:

estimator=wlsmv ;

!LINK=PROBIT ;

PARAMETERIZATION=THETA ;

OUTPUT:

stdyx ;

modindices(10) ;

svalues ;

MODEL:

mem_bnk BY u3* u3 u5 u7 u8 u9 u11 u12 u14 u15 u17 ;

mem_bnk@1 ;

[mem_bnk@0] ;

lmt BY u5* u9 u17 ;

lmt@1 ;

mem_bnk WITH lmt@0 ;

u7 WITH u8* ;

u3 WITH u12* ;

u5 WITH u7* ;

### Step 2

TITLE:

Variable List -

u3 : CERAD immediate sum of 3 trials

u5 : LM immediate

u7 : Brave man immediate

u8 : Brave man delay

u9 : LM delay

u11 : MMSE 3-word delay

u12 : CERAD word list delay

u14 : CERAD constructional praxis delay (MexCog = 4 figures)

u15 : CERAD recognition

u17 : LM recognition

idunique :

dat :

2: 2 LASIDAD

DATA:

FILE = mem_bnk3.dat ;

VARIABLE:

NAMES =

u3 u5 u7 u8 u9 u11 u12 u14 u15 u17 idunique dat ;

MISSING ARE ALL (-9999) ;

idvariable=idunique ;

auxiliary=dat ;

categorical= u11 ;

ANALYSIS:

estimator=wlsmv ;

!LINK=PROBIT ;

PARAMETERIZATION=THETA ;

OUTPUT:

stdyx ;

modindices(10) ;

svalues ;

MODEL:

mem_bnk BY u3* u3 u5 u7 u8 u9 u11 u12 u14 u15 u17 ;

mem_bnk BY u3@4.175399780273438 ;

mem_bnk BY u5@3.541320085525513 ;

mem_bnk BY u7@1.444790005683899 ;

mem_bnk BY u8@2.084709882736206 ;

mem_bnk BY u9@3.904200077056885 ;

mem_bnk BY u11@1.022250056266785 ;

mem_bnk BY u12@2.171600103378296 ;

mem_bnk BY u14@2.121320009231567 ;

mem_bnk BY u15@1.669559955596924 ;

mem_bnk BY u17@1.644260048866272 ;

[u3@17.45351982116699] ;

[u5@9.833490371704102] ;

[u7@7.105010032653809] ;

[u8@5.0901198387146] ;

[u9@7.388929843902588] ;

[u11$1@-2.65327000617981] ;

[u11$2@-1.853819966316223] ;

[u11$3@-.6334499716758728] ;

[u12@5.124229907989502] ;

[u14@5.81043004989624] ;

[u15@18.51020050048828] ;

[u17@10.2783899307251] ;

mem_bnk* ;

[mem_bnk*] ;

u3@9.563000000000001 ;

u5@2.539 ;

u7@3.851 ;

u8@6.55 ;

u9@6.006 ;

u12@2.281 ;

u14@6.007 ;

u15@2.813 ;

u17@4.047 ;

u7 WITH u8@1.785 ;

u3 WITH u12@1.987 ;

u5 WITH u7@1.433 ;

lmt BY u5@3.312 ;

lmt BY u9@2.756 ;

lmt BY u17@0.873 ;

lmt@1 ;

mem_bnk WITH lmt@0 ;

### Step 3

TITLE:

Variable List -

u3 : CERAD immediate sum of 3 trials

u5 : LM immediate

u7 : Brave man immediate

u8 : Brave man delay

u9 : LM delay

u11 : MMSE 3-word delay

u12 : CERAD word list delay

u14 : CERAD constructional praxis delay (MexCog = 4 figures)

u15 : CERAD recognition

u17 : LM recognition

idunique :

dat :

0: 0 HCAP

2: 2 LASIDAD

DATA:

FILE = mem_bnk4.dat ;

VARIABLE:

NAMES =

u3 u5 u7 u8 u9 u11 u12 u14 u15 u17 idunique dat ;

MISSING ARE ALL (-9999) ;

idvariable=idunique ;

auxiliary=dat ;

categorical= u11 ;

ANALYSIS:

estimator=mlr ;

LINK=PROBIT ;

!PARAMETERIZATION=THETA ;

OUTPUT:

stdyx ;

modindices(10) ;

svalues ;

SAVEDATA:

save=fscores ;

file=mem_bnk.dat ;

MODEL:

mem_bnk BY u3@4.175399780273438 ;

mem_bnk BY u5@3.541320085525513 ;

mem_bnk BY u7@1.444790005683899 ;

mem_bnk BY u8@2.084709882736206 ;

mem_bnk BY u9@3.904200077056885 ;

mem_bnk BY u11@1.022250056266785 ;

mem_bnk BY u12@2.171600103378296 ;

mem_bnk BY u14@2.121320009231567 ;

mem_bnk BY u15@1.669559955596924 ;

mem_bnk BY u17@1.644260048866272 ;

[u3@17.45351982116699] ;

[u5@9.833490371704102] ;

[u7@7.105010032653809] ;

[u8@5.0901198387146] ;

[u9@7.388929843902588] ;

[u11$1@-2.65327000617981] ;

[u11$2@-1.853819966316223] ;

[u11$3@-.6334499716758728] ;

[u12@5.124229907989502] ;

[u14@5.81043004989624] ;

[u15@18.51020050048828] ;

[u17@10.2783899307251] ;

mem_bnk@1 ;

[mem_bnk@0] ;

u3@9.563000000000001 ;

u5@2.539 ;

u7@3.851 ;

u8@6.55 ;

u9@6.006 ;

u12@2.281 ;

u14@6.007 ;

u15@2.813 ;

u17@4.047 ;

u7 WITH u8@1.785 ;

u3 WITH u12@1.987 ;

u5 WITH u7@1.433 ;

lmt BY u5@3.312 ;

lmt BY u9@2.756 ;

lmt BY u17@0.873 ;

lmt@1 ;

mem_bnk WITH lmt@0 ;

## Memory factor: DIF adjusted

Mplus input files for each of the following models (item banking approach):

mem_bnk1dif - Memory CFA in HRS/HCAP (step 1)

mem_bnk3dif - Memory CFA in LASI-DAD (step 2)

mem_bnk4dif - Memory CFA in full sample (step 4) - 0 parameters are estimated, factor scores are estimated from this model

### Step 1

### TITLE:

### Variable List -

### u0u3 : CERAD immediate sum of 3 trials-HRS

### u0u5 : LM immediate-HRS

### u7 : Brave man immediate

### u8 : Brave man delay

### u9 : LM delay

### u0u11 : MMSE 3-word delay-HRS

### u12 : CERAD word list delay

### u0u17 : LM recognition-HRS

### u0u14 : CERAD constructional praxis delay (MexCog = 4 figures)-HRS

### u0u15 : CERAD recognition-HRS

### idunique :

### dat :

### 0: 0 HCAP

###

### DATA:

### FILE = mem_bnk1dif.dat ;

### VARIABLE:

### NAMES =

### u0u3 u0u5 u7 u8 u9 u0u11 u12 u0u17 u0u14 u0u15 idunique dat ;

### MISSING ARE ALL (-9999) ;

### idvariable=idunique ;

### auxiliary=dat ;

### categorical= u0u11 ;

###

### ANALYSIS:

### estimator=wlsmv ;

### !LINK=PROBIT ;

### PARAMETERIZATION=THETA ;

###

### OUTPUT:

### stdyx ;

### modindices(10) ;

### svalues ;

###

### MODEL:

### mem_bnk BY u0u3* u0u3 u0u5 u7 u8 u9 u0u11 u12 u0u17 u0u14 u0u15 ;

### mem_bnk@1 ;

### [mem_bnk@0] ;

### lmt BY u0u5* u9 u0u17 ;

### lmt@1 ;

### mem_bnk WITH lmt@0 ;

### u7 WITH u8* ;

### u0u3 WITH u12* ;

### u0u5 WITH u7* ;

### Step 2

TITLE:

Variable List -

u2u3 : CERAD immediate sum of 3 trials-LASIDAD

u2u5 : LM immediate-LASIDAD

u7 : Brave man immediate

u8 : Brave man delay

u9 : LM delay

u2u11 : MMSE 3-word delay-LASIDAD

u12 : CERAD word list delay

u2u17 : LM recognition-LASIDAD

u2u14 : CERAD constructional praxis delay (MexCog = 4 figures)-LASIDAD

u2u15 : CERAD recognition-LASIDAD

idunique :

dat :

2: 2 LASIDAD

DATA:

FILE = mem_bnk3dif.dat ;

VARIABLE:

NAMES =

u2u3 u2u5 u7 u8 u9 u2u11 u12 u2u17 u2u14 u2u15 idunique dat ;

MISSING ARE ALL (-9999) ;

idvariable=idunique ;

auxiliary=dat ;

categorical= u2u11 ;

ANALYSIS:

estimator=wlsmv ;

!LINK=PROBIT ;

PARAMETERIZATION=THETA ;

OUTPUT:

stdyx ;

modindices(10) ;

svalues ;

MODEL:

mem_bnk BY u2u3* u2u3 u2u5 u7 u8 u9 u2u11 u12 u2u17 u2u14 u2u15 ;

mem_bnk BY u7@1.444779992103577 ;

mem_bnk BY u8@2.084709882736206 ;

mem_bnk BY u9@3.90418004989624 ;

mem_bnk BY u12@2.171639919281006 ;

[u7@7.105040073394775] ;

[u8@5.090129852294922] ;

[u9@7.388510227203369] ;

[u12@5.124259948730469] ;

mem_bnk* ;

[mem_bnk*] ;

u2u3@9.561 ;

u2u5@2.536 ;

u7@3.851 ;

u8@6.551 ;

u9@6.005 ;

u12@2.28 ;

u2u17@4.047 ;

u2u14@6.007 ;

u2u15@2.813 ;

u7 WITH u8@1.785 ;

u2u3 WITH u12@1.986 ;

u7 WITH u2u5@1.433 ;

lmt BY u2u5@3.313 ;

lmt BY u9@2.756 ;

lmt BY u2u17@0.873 ;

lmt@1 ;

mem_bnk WITH lmt@0 ;

### Step 3

TITLE:

Variable List -

u0u3 : CERAD immediate sum of 3 trials-HRS

u0u5 : LM immediate-HRS

u7 : Brave man immediate

u8 : Brave man delay

u9 : LM delay

u0u11 : MMSE 3-word delay-HRS

u12 : CERAD word list delay

u0u17 : LM recognition-HRS

u0u14 : CERAD constructional praxis delay (MexCog = 4 figures)-HRS

u0u15 : CERAD recognition-HRS

u2u3 : CERAD immediate sum of 3 trials-LASIDAD

u2u5 : LM immediate-LASIDAD

u2u11 : MMSE 3-word delay-LASIDAD

u2u17 : LM recognition-LASIDAD

u2u14 : CERAD constructional praxis delay (MexCog = 4 figures)-LASIDAD

u2u15 : CERAD recognition-LASIDAD

idunique :

dat :

0: 0 HCAP

2: 2 LASIDAD

DATA:

FILE = mem_bnk4dif.dat ;

VARIABLE:

NAMES =

u0u3 u0u5 u7 u8 u9 u0u11 u12 u0u17 u0u14 u0u15 u2u3 u2u5 u2u11

u2u17 u2u14 u2u15 idunique dat ;

MISSING ARE ALL (-9999) ;

idvariable=idunique ;

auxiliary=dat ;

categorical= u0u11 u2u11 ;

ANALYSIS:

estimator=mlr ;

LINK=PROBIT ;

!PARAMETERIZATION=THETA ;

OUTPUT:

stdyx ;

modindices(10) ;

svalues ;

SAVEDATA:

save=fscores ;

file=mem_bnk.dat ;

MODEL:

mem_bnk BY u0u3@4.175489902496338 ;

mem_bnk BY u0u5@3.541100025177002 ;

mem_bnk BY u7@1.444779992103577 ;

mem_bnk BY u8@2.084709882736206 ;

mem_bnk BY u9@3.90418004989624 ;

mem_bnk BY u0u11@1.022250056266785 ;

mem_bnk BY u12@2.171639919281006 ;

mem_bnk BY u0u17@1.644219994544983 ;

mem_bnk BY u0u14@2.121340036392212 ;

mem_bnk BY u0u15@1.669569969177246 ;

mem_bnk BY u2u3@4.846489906311035 ;

mem_bnk BY u2u5@2.796040058135986 ;

mem_bnk BY u2u11@.7249400019645691 ;

mem_bnk BY u2u17@2.152829885482788 ;

mem_bnk BY u2u14@1.695150017738342 ;

mem_bnk BY u2u15@3.060849905014038 ;

[u0u3@17.45327949523926] ;

[u0u5@9.833430290222168] ;

[u7@7.105040073394775] ;

[u8@5.090129852294922] ;

[u9@7.388510227203369] ;

[u0u11$1@-2.653280019760132] ;

[u0u11$2@-1.853819966316223] ;

[u0u11$3@-.6334599852561951] ;

[u12@5.124259948730469] ;

[u0u17@10.27842044830322] ;

[u0u14@5.810410022735596] ;

[u0u15@18.51021957397461] ;

[u2u3@16.35346031188965] ;

[u2u5@6.889110088348389] ;

[u2u11$1@-1.966050028800964] ;

[u2u11$2@-1.307559967041016] ;

[u2u11$3@-.4270699918270111] ;

[u2u17@9.909870147705078] ;

[u2u14@4.443210124969482] ;

[u2u15@19.12014961242676] ;

mem_bnk BY u7@1.444779992103577 ;

mem_bnk BY u8@2.084709882736206 ;

mem_bnk BY u9@3.90418004989624 ;

mem_bnk BY u12@2.171639919281006 ;

[u7@7.105040073394775] ;

[u8@5.090129852294922] ;

[u9@7.388510227203369] ;

[u12@5.124259948730469] ;

mem_bnk@1 ;

[mem_bnk@0] ;

u0u3@9.561 ;

u0u5@2.536 ;

u7@3.851 ;

u8@6.551 ;

u9@6.005 ;

u12@2.28 ;

u0u17@4.047 ;

u0u14@6.007 ;

u0u15@2.813 ;

u2u3@9.561 ;

u2u5@2.536 ;

u7@3.851 ;

u8@6.551 ;

u9@6.005 ;

u12@2.28 ;

u2u17@4.047 ;

u2u14@6.007 ;

u2u15@2.813 ;

u7 WITH u8@1.785 ;

u0u3 WITH u12@1.986 ;

u7 WITH u0u5@1.433 ;

lmt BY u0u5@3.313 ;

lmt BY u9@2.756 ;

lmt BY u0u17@0.873 ;

lmt@1 ;

mem_bnk WITH lmt@0 ;

u7 WITH u8@1.785 ;

u2u3 WITH u12@1.986 ;

u7 WITH u2u5@1.433 ;

lmt BY u2u5@3.313 ;

lmt BY u9@2.756 ;

lmt BY u2u17@0.873 ;

lmt@1 ;

mem_bnk WITH lmt@0 ;

# Language domain

Mplus input files for each of the following models (item banking approach):

lan_bnk1 - Language CFA in HRS/HCAP (step 1)

lan_bnk3 - Language CFA in LASI-DAD literate (step 2)

lan_bnk4 - Language CFA in LASI-DAD illiterate (step 3)

lan_bnk5 - Language CFA in full sample (step 4) - 0 parameters are estimated, factor scores are estimated from this model

## Language factor: Non-DIF adjusted

### Step 1

TITLE:

Variable List -

u39 : Animal fluency

u40 : TICS Name cactus

u42 : TICS Name scissors

u43 : MMSE naming (watch)

u44 : MMSE naming (pencil)

u46 : Name elbow

u47 : MMSE write (your name?) a sentence

u49 : MMSE read and follow command (closeyoureyes in HCAP)

u51 : Repetition of phrase (noifsandsorbuts in HCAP)

u52 : Do with a hammer

u54 : following instructions 2 step (point to window and door in HCAP)

u55 : Where is the local market?

u56 : following instructions 3 step (paper, fold, floor) (point, sky, ground)

idunique :

dat :

0: 0 HCAP

DATA:

FILE = lan_bnk1.dat ;

VARIABLE:

NAMES =

u39 u40 u42 u43 u44 u46 u47 u49 u51 u52 u54 u55 u56 idunique dat ;

MISSING ARE ALL (-9999) ;

idvariable=idunique ;

auxiliary=dat ;

categorical= u40 u42 u43 u44 u46 u47 u49 u51 u52 u54 u55 u56 ;

ANALYSIS:

estimator=wlsmv ;

!LINK=PROBIT ;

PARAMETERIZATION=THETA ;

OUTPUT:

stdyx ;

modindices(10) ;

svalues ;

MODEL:

lan_bnk BY u39* u39 u40 u42 u43 u44 u46 u47 u49 u51 u52 u54 u55 u56 ;

lan_bnk@1 ;

[lan_bnk@0] ;

### Step 2

TITLE:

Variable List -

u39 : Animal fluency

u41 : TICS name coconut

u42 : TICS Name scissors

u43 : MMSE naming (watch)

u44 : MMSE naming (pencil)

u46 : Name elbow

u47 : MMSE write (your name?) a sentence

u49 : MMSE read and follow command (closeyoureyes in HCAP)

u51 : Repetition of phrase (noifsandsorbuts in HCAP)

u52 : Do with a hammer

u54 : following instructions 2 step (point to window and door in HCAP)

u55 : Where is the local market?

u56 : following instructions 3 step (paper, fold, floor) (point, sky, ground)

idunique :

dat :

2: 2 LASIDAD

DATA:

FILE = lan_bnk3.dat ;

VARIABLE:

NAMES =

u39 u41 u42 u43 u44 u46 u47 u49 u51 u52 u54 u55 u56 idunique dat ;

MISSING ARE ALL (-9999) ;

idvariable=idunique ;

auxiliary=dat ;

categorical= u41 u42 u43 u44 u46 u47 u49 u51 u52 u54 u55 u56 ;

ANALYSIS:

estimator=wlsmv ;

!LINK=PROBIT ;

PARAMETERIZATION=THETA ;

OUTPUT:

stdyx ;

modindices(10) ;

svalues ;

MODEL:

lan_bnk BY u39* u39 u41 u42 u43 u44 u46 u47 u49 u51 u52 u54 u55 u56 ;

lan_bnk BY u39@3.737610101699829 ;

lan_bnk BY u42@.6684600114822388 ;

lan_bnk BY u43@1.303239941596985 ;

lan_bnk BY u44@.892579972743988 ;

lan_bnk BY u46@1.480039954185486 ;

lan_bnk BY u47@.711080014705658 ;

lan_bnk BY u49@1.54069995880127 ;

lan_bnk BY u51@.5120999813079834 ;

lan_bnk BY u52@.3563399910926819 ;

lan_bnk BY u54@1.366170048713684 ;

lan_bnk BY u55@.4753400087356567 ;

lan_bnk BY u56@.6692000031471252 ;

[u39@15.96866035461426] ;

[u42$1@-2.66198992729187] ;

[u43$1@-4.255340099334717] ;

[u44$1@-3.30088996887207] ;

[u46$1@-4.344200134277344] ;

[u47$1@-1.915429949760437] ;

[u49$1@-3.546169996261597] ;

[u51$1@-.6029599905014038] ;

[u52$1@-1.547580003738403] ;

[u54$1@-3.98523998260498] ;

[u55$1@-1.079660058021545] ;

[u56$1@-3.753629922866821] ;

[u56$2@-2.587970018386841] ;

[u56$3@-.9088799953460693] ;

lan_bnk* ;

[lan_bnk*] ;

### Step 3

TITLE:

Variable List -

u39 : Animal fluency

u41 : TICS name coconut

u42 : TICS Name scissors

u43 : MMSE naming (watch)

u44 : MMSE naming (pencil)

u46 : Name elbow

u48 : Say a sentence

u50 : follow example (close your eyes)

u51 : Repetition of phrase (noifsandsorbuts in HCAP)

u52 : Do with a hammer

u54 : following instructions 2 step (point to window and door in HCAP)

u55 : Where is the local market?

u56 : following instructions 3 step (paper, fold, floor) (point, sky, ground)

idunique :

dat :

2: 2 LASIDAD

DATA:

FILE = lan_bnk4.dat ;

VARIABLE:

NAMES =

u39 u41 u42 u43 u44 u46 u48 u50 u51 u52 u54 u55 u56 idunique dat ;

MISSING ARE ALL (-9999) ;

idvariable=idunique ;

auxiliary=dat ;

categorical= u41 u42 u43 u44 u46 u48 u50 u51 u52 u54 u55 u56 ;

ANALYSIS:

estimator=wlsmv ;

!LINK=PROBIT ;

PARAMETERIZATION=THETA ;

OUTPUT:

stdyx ;

modindices(10) ;

svalues ;

MODEL:

lan_bnk BY u39* u39 u41 u42 u43 u44 u46 u48 u50 u51 u52 u54 u55 u56 ;

lan_bnk BY u39@3.737610101699829 ;

lan_bnk BY u41@.5437600016593933 ;

lan_bnk BY u42@.6684600114822388 ;

lan_bnk BY u43@1.303239941596985 ;

lan_bnk BY u44@.892579972743988 ;

lan_bnk BY u46@1.480039954185486 ;

lan_bnk BY u51@.5120999813079834 ;

lan_bnk BY u52@.3563399910926819 ;

lan_bnk BY u54@1.366170048713684 ;

lan_bnk BY u55@.4753400087356567 ;

lan_bnk BY u56@.6692000031471252 ;

[u39@15.96866035461426] ;

[u41$1@-1.299090027809143] ;

[u42$1@-2.66198992729187] ;

[u43$1@-4.255340099334717] ;

[u44$1@-3.30088996887207] ;

[u46$1@-4.344200134277344] ;

[u51$1@-.6029599905014038] ;

[u52$1@-1.547580003738403] ;

[u54$1@-3.98523998260498] ;

[u55$1@-1.079660058021545] ;

[u56$1@-3.753629922866821] ;

[u56$2@-2.587970018386841] ;

[u56$3@-.9088799953460693] ;

lan_bnk* ;

[lan_bnk*] ;

### Step 4

TITLE:

Variable List -

u39 : Animal fluency

u40 : TICS Name cactus

u41 : TICS name coconut

u42 : TICS Name scissors

u43 : MMSE naming (watch)

u44 : MMSE naming (pencil)

u46 : Name elbow

u47 : MMSE write (your name?) a sentence

u48 : Say a sentence

u49 : MMSE read and follow command (closeyoureyes in HCAP)

u50 : follow example (close your eyes)

u51 : Repetition of phrase (noifsandsorbuts in HCAP)

u52 : Do with a hammer

u54 : following instructions 2 step (point to window and door in HCAP)

u55 : Where is the local market?

u56 : following instructions 3 step (paper, fold, floor) (point, sky, ground)

idunique :

dat :

0: 0 HCAP

2: 2 LASIDAD

DATA:

FILE = lan_bnk5.dat ;

VARIABLE:

NAMES =

u39 u40 u41 u42 u43 u44 u46 u47 u48 u49 u50 u51 u52 u54 u55 u56

idunique dat ;

MISSING ARE ALL (-9999) ;

idvariable=idunique ;

auxiliary=dat ;

categorical= u40 u41 u42 u43 u44 u46 u47 u48 u49 u50 u51 u52 u54

u55 u56 ;

ANALYSIS:

estimator=MLR ;

LINK=PROBIT ;

!PARAMETERIZATION=THETA ;

OUTPUT:

stdyx ;

modindices(10) ;

svalues ;

SAVEDATA:

save=fscores ;

file=lanall.dat ;

MODEL:

lan_bnk BY u39@3.737610101699829 ;

lan_bnk BY u40@.8233799934387207 ;

lan_bnk BY u41@.5437600016593933 ;

lan_bnk BY u42@.6684600114822388 ;

lan_bnk BY u43@1.303239941596985 ;

lan_bnk BY u44@.892579972743988 ;

lan_bnk BY u46@1.480039954185486 ;

lan_bnk BY u47@.711080014705658 ;

lan_bnk BY u48@1.186190009117126 ;

lan_bnk BY u49@1.54069995880127 ;

lan_bnk BY u50@1.270089983940125 ;

lan_bnk BY u51@.5120999813079834 ;

lan_bnk BY u52@.3563399910926819 ;

lan_bnk BY u54@1.366170048713684 ;

lan_bnk BY u55@.4753400087356567 ;

lan_bnk BY u56@.6692000031471252 ;

[u39@15.96866035461426] ;

[u40$1@-2.437910079956055] ;

[u41$1@-1.299090027809143] ;

[u42$1@-2.66198992729187] ;

[u43$1@-4.255340099334717] ;

[u44$1@-3.30088996887207] ;

[u46$1@-4.344200134277344] ;

[u47$1@-1.915429949760437] ;

[u48$1@-3.226560115814209] ;

[u49$1@-3.546169996261597] ;

[u50$1@-3.396909952163696] ;

[u51$1@-.6029599905014038] ;

[u52$1@-1.547580003738403] ;

[u54$1@-3.98523998260498] ;

[u55$1@-1.079660058021545] ;

[u56$1@-3.753629922866821] ;

[u56$2@-2.587970018386841] ;

[u56$3@-.9088799953460693] ;

u39@29.189 ;

lan_bnk@1 ;

[lan_bnk@0] ;

## Language factor: DIF adjusted

Mplus input files for each of the following models (item banking approach):

lan_bnk1 - Language CFA in HRS/HCAP (step 1)

lan_bnk3 - Language CFA in LASI-DAD literate (step 2)

lan_bnk4 - Language CFA in LASI-DAD illiterate (step 3)

lan_bnk5 - Language CFA in full sample (step 4) - 0 parameters are estimated, factor scores are estimated from this model

### Step 1

TITLE:

Variable List -

u39 : Animal fluency

u40 : TICS Name cactus

u0u42 : TICS Name scissors-HRS

u43 : MMSE naming (watch)

u44 : MMSE naming (pencil)

u46 : Name elbow

u0u47 : MMSE write (your name?) a sentence-HRS

u0u49 : MMSE read and follow command (closeyoureyes in HCAP)-HRS

u0u51 : Repetition of phrase (noifsandsorbuts in HCAP)-HRS

u52 : Do with a hammer

u0u54 : following instructions 2 step (point to window and door in HCAP)-HRS

u0u55 : Where is the local market?-HRS

u0u56 : following instructions 3 step (paper, fold, floor) (point, sky, ground)-HRS

idunique :

dat :

0: 0 HCAP

DATA:

FILE = lan_bnk1.dat ;

VARIABLE:

NAMES =

u39 u40 u0u42 u43 u44 u46 u0u47 u0u49 u0u51 u52 u0u54 u0u55 u0u56

idunique dat ;

MISSING ARE ALL (-9999) ;

idvariable=idunique ;

auxiliary=dat ;

categorical= u40 u0u42 u43 u44 u46 u0u47 u0u49 u0u51 u52 u0u54

u0u55 u0u56 ;

ANALYSIS:

estimator=wlsmv ;

!LINK=PROBIT ;

PARAMETERIZATION=THETA ;

OUTPUT:

stdyx ;

modindices(10) ;

svalues ;

MODEL:

lan_bnk BY u39* u39 u40 u0u42 u43 u44 u46 u0u47 u0u49 u0u51 u52

u0u54 u0u55 u0u56 ;

lan_bnk@1 ;

[lan_bnk@0] ;

U0U56 WITH U0U49 ;

### Step 2

TITLE:

Variable List -

u39 : Animal fluency

u41 : TICS name coconut

u2u42 : TICS Name scissors-LASIDAD

u43 : MMSE naming (watch)

u44 : MMSE naming (pencil)

u46 : Name elbow

u2u47 : MMSE write (your name?) a sentence-LASIDAD

u2u49 : MMSE read and follow command (closeyoureyes in HCAP)-LASIDAD

u2u51 : Repetition of phrase (noifsandsorbuts in HCAP)-LASIDAD

u52 : Do with a hammer

u2u54 : following instructions 2 step (point to window and door in HCAP)-LASIDAD

u2u55 : Where is the local market?-LASIDAD

u2u56 : following instructions 3 step (paper, fold, floor) (point, sky, ground)-LASIDAD

idunique :

dat :

2: 2 LASIDAD

DATA:

FILE = lan_bnk3.dat ;

VARIABLE:

NAMES =

u39 u41 u2u42 u43 u44 u46 u2u47 u2u49 u2u51 u52 u2u54 u2u55 u2u56

idunique dat ;

MISSING ARE ALL (-9999) ;

idvariable=idunique ;

auxiliary=dat ;

categorical= u41 u2u42 u43 u44 u46 u2u47 u2u49 u2u51 u52 u2u54

u2u55 u2u56 ;

ANALYSIS:

estimator=wlsmv ;

!LINK=PROBIT ;

PARAMETERIZATION=THETA ;

OUTPUT:

stdyx ;

modindices(10) ;

svalues ;

MODEL:

lan_bnk BY u39* u39 u41 u2u42 u43 u44 u46 u2u47 u2u49 u2u51 u52

u2u54 u2u55 u2u56 ;

lan_bnk BY u39@4.094329833984375 ;

lan_bnk BY u43@1.413300037384033 ;

lan_bnk BY u44@.9614400267601013 ;

lan_bnk BY u46@1.656919956207275 ;

lan_bnk BY u52@.3832699954509735 ;

[u39@15.96905994415283] ;

[u43$1@-4.483719825744629] ;

[u44$1@-3.416369915008545] ;

[u46$1@-4.7082200050354] ;

[u52$1@-1.561139941215515] ;

lan_bnk* ;

[lan_bnk*] ;

### Step 3

TITLE:

Variable List -

u39 : Animal fluency

u41 : TICS name coconut

u2u42 : TICS Name scissors-LASIDAD

u43 : MMSE naming (watch)

u44 : MMSE naming (pencil)

u46 : Name elbow

u48 : Say a sentence

u50 : follow example (close your eyes)

u2u51 : Repetition of phrase (noifsandsorbuts in HCAP)-LASIDAD

u52 : Do with a hammer

u2u54 : following instructions 2 step (point to window and door in HCAP)-LASIDAD

u2u55 : Where is the local market?-LASIDAD

u2u56 : following instructions 3 step (paper, fold, floor) (point, sky, ground)-LASIDAD

idunique :

dat :

2: 2 LASIDAD

DATA:

FILE = lan_bnk4.dat ;

VARIABLE:

NAMES =

u39 u41 u2u42 u43 u44 u46 u48 u50 u2u51 u52 u2u54 u2u55 u2u56

idunique dat ;

MISSING ARE ALL (-9999) ;

idvariable=idunique ;

auxiliary=dat ;

categorical= u41 u2u42 u43 u44 u46 u48 u50 u2u51 u52 u2u54 u2u55

u2u56 ;

ANALYSIS:

estimator=wlsmv ;

!LINK=PROBIT ;

PARAMETERIZATION=THETA ;

OUTPUT:

stdyx ;

modindices(10) ;

svalues ;

MODEL:

lan_bnk BY u39* u39 u41 u2u42 u43 u44 u46 u48 u50 u2u51 u52 u2u54

u2u55 u2u56 ;

lan_bnk BY u39@4.094329833984375 ;

lan_bnk BY u41@.7430300116539001 ;

lan_bnk BY u2u42@.4744400084018707 ;

lan_bnk BY u43@1.413300037384033 ;

lan_bnk BY u44@.9614400267601013 ;

lan_bnk BY u46@1.656919956207275 ;

lan_bnk BY u2u51@.7141500115394592 ;

lan_bnk BY u52@.3832699954509735 ;

lan_bnk BY u2u54@1.268120050430298 ;

lan_bnk BY u2u55@.7902100086212158 ;

lan_bnk BY u2u56@.6179599761962891 ;

[u39@15.96905994415283] ;

[u41$1@-1.356040000915527] ;

[u2u42$1@-1.695440053939819] ;

[u43$1@-4.483719825744629] ;

[u44$1@-3.416369915008545] ;

[u46$1@-4.7082200050354] ;

[u2u51$1@-2.540560007095337] ;

[u52$1@-1.561139941215515] ;

[u2u54$1@-3.14913010597229] ;

[u2u55$1@-2.65831995010376] ;

[u2u56$1@-3.312259912490845] ;

[u2u56$2@-2.564100027084351] ;

[u2u56$3@-1.653679966926575] ;

lan_bnk* ;

[lan_bnk*] ;

### Step 4

TITLE:

Variable List -

u39 : Animal fluency

u40 : TICS Name cactus

u41 : TICS name coconut

u0u42 : TICS Name scissors-HRS

u43 : MMSE naming (watch)

u44 : MMSE naming (pencil)

u46 : Name elbow

u0u47 : MMSE write (your name?) a sentence-HRS

u0u49 : MMSE read and follow command (closeyoureyes in HCAP)-HRS

u0u51 : Repetition of phrase (noifsandsorbuts in HCAP)-HRS

u52 : Do with a hammer

u0u54 : following instructions 2 step (point to window and door in HCAP)-HRS

u0u55 : Where is the local market?-HRS

u0u56 : following instructions 3 step (paper, fold, floor) (point, sky, ground)-HRS

u2u42 : TICS Name scissors-LASIDAD

u2u47 : MMSE write (your name?) a sentence-LASIDAD

u2u49 : MMSE read and follow command (closeyoureyes in HCAP)-LASIDAD

u2u51 : Repetition of phrase (noifsandsorbuts in HCAP)-LASIDAD

u2u54 : following instructions 2 step (point to window and door in HCAP)-LASIDAD

u2u55 : Where is the local market?-LASIDAD

u2u56 : following instructions 3 step (paper, fold, floor) (point, sky, ground)-LASIDAD

u48 : Say a sentence

u50 : follow example (close your eyes)

idunique :

dat :

0: 0 HCAP

2: 2 LASIDAD

DATA:

FILE = lan_bnk5.dat ;

VARIABLE:

NAMES =

u39 u40 u41 u0u42 u43 u44 u46 u0u47 u0u49 u0u51 u52 u0u54 u0u55

u0u56 u2u42 u2u47 u2u49 u2u51 u2u54 u2u55 u2u56 u48 u50 idunique

dat ;

MISSING ARE ALL (-9999) ;

idvariable=idunique ;

auxiliary=dat ;

categorical= u40 u41 u0u42 u43 u44 u46 u0u47 u0u49 u0u51 u52

u0u54 u0u55 u0u56 u2u42 u2u47 u2u49 u2u51 u2u54 u2u55 u2u56 u48

u50 ;

ANALYSIS:

estimator=MLR ;

LINK=PROBIT ;

!PARAMETERIZATION=THETA ;

OUTPUT:

stdyx ;

modindices(10) ;

svalues ;

SAVEDATA:

save=fscores ;

file=lanall.dat ;

MODEL:

lan_bnk BY u39@4.094329833984375 ;

lan_bnk BY u40@.9004499912261963 ;

lan_bnk BY u41@.7430300116539001 ;

lan_bnk BY u0u42@.7061100006103516 ;

lan_bnk BY u43@1.413300037384033 ;

lan_bnk BY u44@.9614400267601013 ;

lan_bnk BY u46@1.656919956207275 ;

lan_bnk BY u0u47@.7539899945259094 ;

lan_bnk BY u0u49@.606939971446991 ;

lan_bnk BY u0u51@.5440300107002258 ;

lan_bnk BY u52@.3832699954509735 ;

lan_bnk BY u0u54@1.448590040206909 ;

lan_bnk BY u0u55@.500249981880188 ;

lan_bnk BY u0u56@.4008600115776062 ;

lan_bnk BY u2u42@.4744400084018707 ;

lan_bnk BY u2u47@1.068379998207092 ;

lan_bnk BY u2u49@.6002900004386902 ;

lan_bnk BY u2u51@.7141500115394592 ;

lan_bnk BY u2u54@1.268120050430298 ;

lan_bnk BY u2u55@.7902100086212158 ;

lan_bnk BY u2u56@.6179599761962891 ;

lan_bnk BY u48@1.39028000831604 ;

lan_bnk BY u50@1.518499970436096 ;

[u39@15.96905994415283] ;

[u40$1@-2.532789945602417] ;

[u41$1@-1.356040000915527] ;

[u0u42$1@-2.708790063858032] ;

[u43$1@-4.483719825744629] ;

[u44$1@-3.416369915008545] ;

[u46$1@-4.7082200050354] ;

[u0u47$1@-1.955019950866699] ;

[u0u49$1@-2.258539915084839] ;

[u0u51$1@-.611020028591156] ;

[u52$1@-1.561139941215515] ;

[u0u54$1@-4.143640041351318] ;

[u0u55$1@-1.090240001678467] ;

[u0u56$1@-3.359780073165894] ;

[u0u56$2@-2.317169904708862] ;

[u0u56$3@-.8137900233268738] ;

[u2u42$1@-1.695440053939819] ;

[u2u47$1@-2.841130018234253] ;

[u2u49$1@-.4059000015258789] ;

[u2u51$1@-2.540560007095337] ;

[u2u54$1@-3.14913010597229] ;

[u2u55$1@-2.65831995010376] ;

[u2u56$1@-3.312259912490845] ;

[u2u56$2@-2.564100027084351] ;

[u2u56$3@-1.653679966926575] ;

[u48$1@-3.7051100730896] ;

[u50$1@-3.973789930343628] ;

u39@26.393 ;

lan_bnk@1 ;

[lan_bnk@0] ;
