# Supplementary material for: Cross-national harmonization of cognitive measures across HRS HCAP (USA) and LASI-DAD (India)
Source: PLoS One. 2022 Feb 25;17(2):e0264166. doi: 10.1371/journal.pone.0264166 (PMC8880818; doi:10.1371/journal.pone.0264166)
Supplement: S2 File — (DOCX) [file pone.0264166.s002.docx]

# Supplement B. Stata syntax for DIF analyses with regression approach

## Memory domain

* install required programs

ssc install runmplus

* write a program to use Mplus to generate factor score estimates

cap program drop fscoreme

program define fscoreme, rclass

syntax varlist, idvar(string) factor(string)

local cat ""

foreach x in `varlist' {

qui distinct `x'

if `r(ndistinct)'<=10 {

local cat "`cat' `x'"

}

}

local w1: word 1 of `varlist'

runmplus `varlist' `idvar' , ///dat

variable(idvariable=`idvar'; categorical=`cat'; ) ///

analysis(estimator=MLR; ) ///

model(`factor' BY `w1'* `varlist'; `factor'@1; [`factor'@0]; ) ///

output(stdyx; modindices(10); ) savelogfile( lanomni) savedata(save=fscores ; file=lanomni.dat) ///

saveinputfile(lan_omni) saveinputdatafile(lan_omni)

preserve

qui runmplus_load_savedata , out(c:\trash\lanomni.out) clear

keep `idvar' `factor'*

tempfile foo

qui save `foo'

restore

capture drop _merge

merge `idvar' using `foo', sort update keep(`factor')

drop _merge

end

* write a program to conduct the logistic regression

cap program drop logregme

program define logregme, rclass

syntax varlist , f(string)

local array ""

local arrayx ""

qui foreach x in `varlist' { // `vlistcomm'

*di in red "*********************doing `x'"

qui distinct `x'

if `r(ndistinct)'>10 {

local oper "regress"

}

else {

local oper "ologit"

}

`oper' `x' `f' age female raedyrs //

local ll_1`x' = e(ll)

local df_1`x' = e(df_m)

`oper' `x' `f' dat age female raedyrs //

local ll_2`x' = e(ll)

local df_2`x' = e(df_m)

mat udif2`x' = r(table)

foreach p in b se pvalue {

eme `p' , mat(udif2`x') col(2)

local udif`x'`p': di %5.3f `r(r1)'

return local udif`x'`p' = `udif`x'`p''

}

*Uniform DIF

local chi2udif`x' = abs(-2*`ll_1`x'' + 2*`ll_2`x'') local array "`array' `chi2udif`x'',"

local arrayx "`arrayx' `x'=`chi2udif`x'',"

}

local array "`array' 0"

di in white "<<`arrayx'>>"

return local array "`array'"

local foo= max(`array' )

di in white "??`foo'??"

return local maxll = `foo'

* which of varlist caused that `maxll'?

foreach x in `varlist' {

if `chi2udif`x''==`foo' {

return local var "`x'"

}

}

end

use $derived/interim-fh2019harm-120.dta, clear

rename data dat

drop if dat==1

replace dat=1 if dat==2 // required to interpret the regressions.

label define dat 0 "0 HCAP" 1 "1 LASIDAD"

label values dat dat

tab dat

local vlist "u3 u5 u7 u8 u9 u11 u12 u14 u15 u17 "

local vlistcomm "u3 u5 u7 u8 u9 u11 u12 u14 u15 u17 "

local vlistncom " "

sum `vlist'

di invchi2(1,1-.1030147/2) //returns the chi2

local p=0.05/(12*12*2 + 10*10*2) // 12 variables, 12 steps in, and 2 approaches (log reg, alignment). And 10 variables for memory

di invchi2(1,1-`p'/2) //returns the chi2

local chi2sign = 42

foreach x in 0 1 2 3 4 5 6 7 8 9 10 {

global var`x' ""

global maxll`x' ""

}

foreach var in `vlistcomm' {

foreach p in b se pvalue {

global udif`var'`p' ""

}

}

qui fscoreme `vlist' , idvar(idunique) factor(f0)

logregme `vlistcomm' , f(f0) // ROUND 1

local maxll = `r(maxll)'

global maxll0 = `r(maxll)'

local array "`r(array)'"

local var "`r(var)'"

global var0 "`r(var)'"

foreach v in `vlistcomm' {

foreach p in b se pvalue {

global udif`v'`p' "`r(udif`v'`p')'"

}

}

local counter=0

while `maxll' >`chi2sign' {

nois di in red "doing `var' **************"

cap drop `var'_0

gen `var'_0=`var' if dat==0

cap drop `var'_1

gen `var'_1=`var' if dat==1

lstrfun vlistcomm , subinstr("`vlistcomm'","`var' ","",.)

local vlistncom "`vlistncom' `var'_0 `var'_1"

qui fscoreme `vlistcomm' `vlistncom', idvar(idunique) factor(f`++counter')

logregme `vlistcomm' , f(f`counter') // ROUND X

local maxll = `r(maxll)'

global maxll`counter' = `r(maxll)'

local array "`r(array)'"

local var "`r(var)'"

global var`counter' "`r(var)'"

foreach v in `vlistcomm' {

foreach p in b se pvalue {

global udif`v'`p' "`r(udif`v'`p')'"

}

}

}

foreach x in 0 1 2 3 4 5 6 7 8 9 10 {

*di in white "${var`x'}, log likelihood = ${maxll`x'}"

local var "${var`x'}"

di in yellow "${var`x'}: b=${udif`var'b}, se=${udif`var'se}, p=${udif`var'pvalue}, log likelihood = ${maxll`x'}"

}

foreach var in `vlistcomm' {

di in yellow "`var': b=${udif`var'b}, se=${udif`var'se}, p=${udif`var'pvalue}"

}

di "final array: `array'"

di "common set: `vlistcomm'"

di "noncom set: `vlistncom'"

describe `vlistcomm'

## Language domain

* Assumes the same programs as for the memory domain have been installed or defined.

*use $derived/interim-fh2019harm-406.dta , clear

use $derived/interim-fh2019harm-120.dta, clear

replace raedyrs=17 if inrange(raedyrs,17, 33)==1

rename data dat

drop if dat==1

replace dat=1 if dat==2 // required to interpret the regressions.

label define dat 0 "0 HCAP" 1 "1 LASIDAD"

label values dat dat

tab dat

local vlist "u39 u40 u41 u42 u43 u44 u46 u47 u48 u49 u50 u51 u52 u54 u55 u56"

local vlistcomm "u39 u42 u43 u44 u46 u47 u49 u51 u52 u54 u55 u56 "

local vlistncom "u40 u41 u48 u50"

sum `vlist'

di chi2(3.84,1) // p=0.1

di invchi2(1,1-.1030147/2) //returns the chi2

local p=0.05/(12*12*2 + 10*10*2) // 12 variables, 12 steps in, and 2 approaches (log reg, alignment). And 10 variables for memory

di invchi2(1,1-`p'/2) //returns the chi2

local chi2sign = 42

foreach x in 0 1 2 3 4 5 6 7 8 9 10 {

global var`x' ""

global maxll`x' ""

}

foreach var in `vlistcomm' {

foreach p in b se pvalue {

global udif`var'`p' ""

}

}

qui fscoreme `vlist' , idvar(idunique) factor(f0)

logregme `vlistcomm' , f(f0) // ROUND 1

local maxll = `r(maxll)'

global maxll0 = `r(maxll)'

local array "`r(array)'"

local var "`r(var)'"

global var0 "`r(var)'"

foreach v in `vlistcomm' {

foreach p in b se pvalue {

global udif`v'`p' "`r(udif`v'`p')'"

}

}

local counter=0

while `maxll' >`chi2sign' {

nois di in red "doing `var' **************"

cap drop `var'_0

gen `var'_0=`var' if dat==0

cap drop `var'_1

gen `var'_1=`var' if dat==1

lstrfun vlistcomm , subinstr("`vlistcomm'","`var' ","",.)

local vlistncom "`vlistncom' `var'_0 `var'_1"

qui fscoreme `vlistcomm' `vlistncom', idvar(idunique) factor(f`++counter')

logregme `vlistcomm' , f(f`counter') // ROUND X

local maxll = `r(maxll)'

global maxll`counter' = `r(maxll)'

local array "`r(array)'"

local var "`r(var)'"

global var`counter' "`r(var)'"

foreach v in `vlistcomm' {

foreach p in b se pvalue {

global udif`v'`p' "`r(udif`v'`p')'"

}

}

}

foreach x in 0 1 2 3 4 5 6 7 8 9 10 {

*di in white "${var`x'}, log likelihood = ${maxll`x'}"

local var "${var`x'}"

di in yellow "${var`x'}: b=${udif`var'b}, se=${udif`var'se}, p=${udif`var'pvalue}, log likelihood = ${maxll`x'}"

}

foreach var in `vlistcomm' {

di in yellow "`var': b=${udif`var'b}, se=${udif`var'se}, p=${udif`var'pvalue}"

}

di "final array: `array'"

di "common set: `vlistcomm'"

di "noncom set: `vlistncom'"

describe `vlistcomm'
