# Supplementary material for: Cross-national harmonization of cognitive measures across HRS HCAP (USA) and LASI-DAD (India)
Source: PLoS One. 2022 Feb 25;17(2):e0264166. doi: 10.1371/journal.pone.0264166 (PMC8880818; doi:10.1371/journal.pone.0264166)
Supplement: S3 File — (DOCX) [file pone.0264166.s003.docx]

# S3 File. Correlation matrices of items for HRS HCAP and LASI-DAD

Supplementary Table 1. Matrix of correlations between items for HRS HCAP

|  |  | 1 | 2 | 3 | 4 | 5 | 6 | 7 | 8 | 9 | 10 | 11 | 12 | 13 | 14 | 15 | 16 | 17 | 18 | 19 | 20 | 21 | 22 |
| --- | --- | --- | --- | --- | --- | --- | --- | --- | --- | --- | --- | --- | --- | --- | --- | --- | --- | --- | --- | --- | --- | --- | --- |
| 1 | Word list immediate recall |  |  |  |  |  |  |  |  |  |  |  |  |  |  |  |  |  |  |  |  |  |  |
| 2 | Word list delayed recall | .919* |  |  |  |  |  |  |  |  |  |  |  |  |  |  |  |  |  |  |  |  |  |
| 3 | Word list recognition | .943* | .938* |  |  |  |  |  |  |  |  |  |  |  |  |  |  |  |  |  |  |  |  |
| 4 | Constructional praxis delayed recall | .841* | .848* | .882* |  |  |  |  |  |  |  |  |  |  |  |  |  |  |  |  |  |  |  |
| 5 | Logical memory immediate recall | .872* | .896* | .896* | .848* |  |  |  |  |  |  |  |  |  |  |  |  |  |  |  |  |  |  |
| 6 | Logical memory delayed recall | .770* | .780* | .785* | .781* | .803* |  |  |  |  |  |  |  |  |  |  |  |  |  |  |  |  |  |
| 7 | Logical memory recognition | .758* | .779* | .774* | .769* | .820* | .979* |  |  |  |  |  |  |  |  |  |  |  |  |  |  |  |  |
| 8 | Brave man immediate recall | .924* | .932* | .956* | .882* | .925* | .812* | .794* |  |  |  |  |  |  |  |  |  |  |  |  |  |  |  |
| 9 | Brave man delayed recall | .775* | .786* | .796* | .792* | .774* | .980* | .959* | .791* |  |  |  |  |  |  |  |  |  |  |  |  |  |  |
| 10 | 3-word delayed recall | .846* | .847* | .840* | .791* | .814* | .736* | .725* | .857* | .737* |  |  |  |  |  |  |  |  |  |  |  |  |  |
| 11 | Animal fluency | .945* | .934* | .952* | .879* | .886* | .778* | .761* | .958* | .794* | .878* |  |  |  |  |  |  |  |  |  |  |  |  |
| 12 | Name cactus | .948* | .937* | .942* | .876* | .889* | .780* | .763* | .954* | .790* | .887* | .990* |  |  |  |  |  |  |  |  |  |  |  |
| 13 | Name scissors | .948* | .937* | .942* | .876* | .889* | .780* | .763* | .954* | .790* | .887* | .990* | 1.000* |  |  |  |  |  |  |  |  |  |  |
| 14 | Name watch | .920* | .909* | .914* | .850* | .863* | .761* | .755* | .920* | .771* | .872* | .954* | .964* | .964* |  |  |  |  |  |  |  |  |  |
| 15 | Name pencil | .920* | .915* | .913* | .855* | .868* | .766* | .760* | .925* | .777* | .872* | .960* | .970* | .970* | .981* |  |  |  |  |  |  |  |  |
| 16 | Name elbow | .939* | .941* | .958* | .885* | .898* | .782* | .771* | .964* | .793* | .860* | .973* | .970* | .970* | .955* | .947* |  |  |  |  |  |  |  |
| 17 | Write a sentence | .743* | .713* | .732* | .721* | .703* | .631* | .634* | .727* | .637* | .696* | .751* | .748* | .748* | .745* | .745* | .734* |  |  |  |  |  |  |
| 18 | Read and follow command | .906* | .877* | .900* | .825* | .837* | .733* | .722* | .900* | .738* | .835* | .927* | .930* | .930* | .928* | .927* | .927* | .774* |  |  |  |  |  |
| 19 | Repetition of phrase | .895* | .884* | .895* | .837* | .844* | .765* | .753* | .900* | .770* | .865* | .928* | .931* | .931* | .916* | .922* | .909* | .743* | .877* |  |  |  |  |
| 20 | What to do with a hammer | .942* | .950* | .961* | .888* | .901* | .791* | .774* | .974* | .801* | .869* | .983* | .980* | .980* | .944* | .950* | .990* | .737* | .917* | .918* |  |  |  |
| 21 | Where is the local market/store? | .942* | .950* | .962* | .882* | .895* | .785* | .774* | .968* | .796* | .863* | .977* | .973* | .973* | .938* | .944* | .983* | .732* | .918* | .912* | .993* |  |  |
| 22 | Following instructions 2 step | .932* | .940* | .958* | .897* | .898* | .788* | .776* | .964* | .804* | .866* | .980* | .970* | .970* | .941* | .947* | .987* | .740* | .921* | .909* | .990* | .983* |  |
| 23 | Following instructions 3 step | .857* | .840* | .851* | .806* | .807* | .710* | .704* | .850* | .715* | .799* | .871* | .880* | .880* | .883* | .877* | .877* | .786* | .933* | .846* | .867* | .868* | .870* |

*Note.* *p<.05

Supplementary Table 2. Matrix of correlations between items for LASI-DAD

|  |  | 1 | 2 | 3 | 4 | 5 | 6 | 7 | 8 | 9 | 10 | 11 | 12 | 13 | 14 | 15 | 16 | 17 | 18 | 19 | 20 | 21 | 22 | 23 | 24 |
| --- | --- | --- | --- | --- | --- | --- | --- | --- | --- | --- | --- | --- | --- | --- | --- | --- | --- | --- | --- | --- | --- | --- | --- | --- | --- |
| 1 | Word list immediate recall | | |  |  |  |  |  |  |  |  |  |  |  |  |  |  |  |  |  |  |  |  |  |  |
| 2 | Word list delayed recall | .174* |  |  |  |  |  |  |  |  |  |  |  |  |  |  |  |  |  |  |  |  |  |  |  |
| 3 | Word list recognition | .575* | .146* |  |  |  |  |  |  |  |  |  |  |  |  |  |  |  |  |  |  |  |  |  |  |
| 4 | Constructional praxis delayed recall | .413* | .111* | .387* |  |  |  |  |  |  |  |  |  |  |  |  |  |  |  |  |  |  |  |  |  |
| 5 | Logical memory immediate recall | .273* | .445* | .253* | .160* |  |  |  |  |  |  |  |  |  |  |  |  |  |  |  |  |  |  |  |  |
| 6 | Logical memory delayed recall | .315* | .377* | .282* | .206* | .613* |  |  |  |  |  |  |  |  |  |  |  |  |  |  |  |  |  |  |  |
| 7 | Logical memory recognition | .295* | .452* | .229* | .158* | .696* | .633* |  |  |  |  |  |  |  |  |  |  |  |  |  |  |  |  |  |  |
| 8 | Brave man immediate recall | .264* | .490* | .220* | .133* | .779* | .552* | .595* |  |  |  |  |  |  |  |  |  |  |  |  |  |  |  |  |  |
| 9 | Brave man delayed recall | .246* | .516* | .168* | .141* | .514* | .631* | .642* | .544* |  |  |  |  |  |  |  |  |  |  |  |  |  |  |  |  |
| 10 | 3-word delayed recall | .159* | .420* | .171* | .079* | .373* | .259* | .348* | .430* | .286* |  |  |  |  |  |  |  |  |  |  |  |  |  |  |  |
| 11 | Animal fluency | 0.002 | 0.017 | 0.01 | 0.006 | 0.022 | 0.016 | 0.017 | 0.013 | 0.014 | .036* |  |  |  |  |  |  |  |  |  |  |  |  |  |  |
| 12 | Name coconut | -0.007 | 0.009 | 0.017 | 0.007 | .040* | 0.029 | 0.029 | 0.006 | 0.011 | 0.003 | .451* |  |  |  |  |  |  |  |  |  |  |  |  |  |
| 13 | Name scissors | -0.009 | 0.001 | 0.007 | 0.001 | 0.027 | .035* | .051* | 0.004 | 0.013 | 0.01 | .494* | .693* |  |  |  |  |  |  |  |  |  |  |  |  |
| 14 | Name watch | -0.031 | 0.001 | -0.023 | 0.007 | 0.006 | 0.01 | 0.02 | -0.014 | 0 | -0.02 | .326* | .446* | .457* |  |  |  |  |  |  |  |  |  |  |  |
| 15 | Name pencil | -0.024 | 0.014 | -0.016 | -0.009 | 0.014 | 0.016 | 0.026 | -0.005 | 0.008 | -0.02 | .337* | .443* | .475* | .881* |  |  |  |  |  |  |  |  |  |  |
| 16 | Name elbow | -0.014 | 0.021 | -0.008 | 0.001 | .035* | 0.029 | .036* | 0.008 | 0.018 | -0.006 | .663* | .489* | .476* | .516* | .512* |  |  |  |  |  |  |  |  |  |
| 17 | Write a sentence | 0.019 | -0.023 | 0.026 | 0.041 | 0.001 | 0.03 | 0.022 | -0.016 | 0.002 | 0.007 | .526* | .560* | .634* | .596* | .621* | .587* |  |  |  |  |  |  |  |  |
| 18 | Say a sentence | -0.01 | -0.008 | -0.022 | 0.021 | .050* | 0.007 | 0.017 | -0.014 | -0.01 | -0.009 | .073* | .341* | .366* | 0.039 | 0.036 | .085* | x |  |  |  |  |  |  |  |
| 19 | Read and follow command | -0.027 | -0.024 | -0.012 | 0.028 | -0.016 | 0.011 | 0.033 | -0.023 | -0.002 | -0.003 | .680* | .723* | .751* | .737* | .767* | .702* | .608* | x |  |  |  |  |  |  |
| 20 | Follow example | -0.024 | -0.013 | -0.04 | -0.037 | 0.01 | -0.003 | -0.009 | -0.024 | -0.016 | -0.015 | .079* | .150* | .138* | .365* | .407* | .350* | x | -0.011 | x |  |  |  |  |  |
| 21 | Repetition of phrase | -.049* | -0.015 | -0.006 | -.035* | -0.016 | -0.013 | -0.004 | -0.032 | -0.009 | -0.014 | .353* | .433* | .432* | .388* | .385* | .382* | .527* | .055* | .656* | .161* |  |  |  |  |
| 22 | What to do with a hammer | -0.024 | 0.019 | -0.014 | 0.005 | 0.033 | 0.026 | 0.034 | 0.007 | 0.016 | -0.007 | .675* | .496* | .527* | .449* | .459* | .876* | .630* | .128* | .702* | .199* | .406* |  |  |  |
| 23 | Where is the local market/store? | -0.028 | 0.017 | -0.006 | -0.01 | 0.029 | 0.027 | 0.029 | 0.004 | 0.013 | -0.008 | .678* | .529* | .511* | .434* | .458* | .849* | .591* | .077* | .708* | .244* | .434* | .882* |  |  |
| 24 | Following instructions 2 step | -0.024 | 0.013 | -0.009 | 0 | .035* | 0.029 | 0.028 | -0.001 | 0.008 | -0.01 | .651* | .484* | .463* | .461* | .471* | .838* | .583* | .151* | .699* | .318* | .397* | .856* | .864* |  |
| 25 | Following instructions 3 step | -0.031 | -0.005 | -0.035 | -0.022 | 0.024 | 0.024 | 0.019 | -0.012 | -0.001 | -0.022 | .419* | .473* | .500* | .598* | .593* | .478* | .625* | .074* | .746* | .313* | .411* | .443* | .453* | .461* |

*Note.* *p<.05; x = Cannot be computed because at least one of the variables is constant.
